# Changes in dietary intake during puberty and their determinants: results from the GINIplus birth cohort study

**DOI:** 10.1186/s12889-015-2189-0

**Published:** 2015-09-02

**Authors:** Carla Harris, Claudia Flexeder, Elisabeth Thiering, Anette Buyken, Dietrich Berdel, Sibylle Koletzko, Carl-Peter Bauer, Irene Brüske, Berthold Koletzko, Marie Standl, for the GINIplus Study Group

**Affiliations:** Institute of Epidemiology I, Helmholtz Zentrum München – German Research Centre for Environmental Health, Ingolstädter Landstr. 1, 85764 Neuherberg, Germany; Ludwig-Maximilians-University of Munich, Dr. von Hauner Children’s Hospital, Munich, Germany; Research Institute of Child Nutrition, University of Bonn, Dortmund, Germany; Department of Pediatrics, Marien-Hospital Wesel, Wesel, Germany; Technical University of Munich, Department of Pediatrics, Munich, Germany

**Keywords:** Puberty, Dietary intake, Dietary changes, Tracking, Determinants, Epidemiology

## Abstract

**Background:**

Understanding changes in dietary intake during puberty could aid the mapping of dietary interventions for primary prevention. The present study describes dietary changes from childhood to adolescence, and their associations with parental education, family income, child education, body mass index (BMI), pubertal onset and screen-time sedentary behaviour.

**Methods:**

Dietary data (*n* = 1232) were obtained from food frequency questionnaires at the 10- and 15-year follow-ups of the GINIplus birth cohort study. Intakes of 17 food groups, macronutrients and antioxidant vitamins, were described by a) paired Wilcoxon rank sum tests, comparing average intakes at each time-point, and b) Cohen’s kappa “tracking” coefficients, measuring stability of intakes (maintenance of relative tertile positions across time). Further, associations of changes (tertile position increase or decrease vs. tracking) with parental education, family income, child education, pubertal onset, BMI, and screen-time, were assessed by logistic regression and multinomial logistic regression models stratified by baseline intake tertile.

**Results:**

Both sexes increased average intakes of water and decreased starchy vegetables, margarine and dairy. Females decreased meat and retinol intakes and increased vegetables, grains, oils and tea. Males decreased fruit and carbohydrates and increased average intakes of meat, caloric drinks, water, protein, fat, polyunsaturated fatty acids (PUFAs), vitamin C and alpha-tocopherol. Both sexes presented mainly “fair” tracking levels [κ_w_ = 0.21–0.40]. Females with high (vs. low) parental education were more likely to increase their nut intake [OR = 3.8; 95 % CI = (1.7;8.8)], and less likely to decrease vitamin C intakes [0.2 (0.1;0.5)], while males were less likely to increase egg consumption [0.2 (0.1;0.5)] and n3 PUFAs [0.2 (0.1;0.5)]. Females with a higher (vs. low) family income were more likely to maintain medium wholegrain intakes [0.2 (0.1;0.7) for decrease vs. tracking, and 0.1 (0.0;0.5) for increase vs. tracking], and were less likely to decrease vitamin C intakes [0.2 (0.1;0.6)]. Males with high education were less likely to increase sugar-sweetened foods [0.1 (0.1;0.4)]. Finally, BMI in females was negatively associated with decreasing protein intakes [0.7 (0.6;0.9)]. In males BMI was positively associated with increasing margarine [1.4 (1.1;1.6)] and vitamin C intakes [1.4 (1.1;1.6)], and negatively associated with increasing n3 PUFA.

**Conclusions:**

Average dietary intakes changed significantly, despite fair tracking levels, suggesting the presence of trends in dietary behaviour during puberty. Family income and parental education predominantly influenced intake changes. Our results support the rationale for dietary interventions targeting children, and suggest that sex-specific subpopulations, e.g. low socio-economic status, should be considered for added impact.

**Electronic supplementary material:**

The online version of this article (doi:10.1186/s12889-015-2189-0) contains supplementary material, which is available to authorized users.

## Background

Public health interventions, aimed at the primary prevention of chronic diseases through diet, typically focus on education and facilitation towards the development of healthier eating habits [1–3]. Children are often targeted, due to the underlying evidence that the physiological risk of chronic diseases can develop early in childhood [4]. However, newly adopted health conducts in children may not be maintained throughout adolescence, as behaviour during this stage is often erratic and prone to changes [5]. Understanding food intake changes during the transition into adolescence can hence help guide the mapping of dietary interventions for primary prevention. Aside from general dietary alterations occurring at the population level, knowledge regarding the stability of individual diet during puberty could help answer questions such as *when* to introduce dietary interventions to ensure optimal adoption and maintenance. Furthermore, evaluating which factors may determine particular dietary changes could help to identify possible subpopulations as important targets for dietary interventions.

The maintenance of food intake behaviour over time, relative to the rest of the population, is referred to as “dietary tracking” [6]. The presence and strength of dietary tracking, or lack thereof, can reflect the level of stability of individual long-term eating behaviours. A 2012 review [7], summarizing the results of studies assessing tracking levels of dietary patterns from childhood to adolescence [8–11], reported weak to moderate tracking of intakes including fruit and vegetables, total energy, macronutrients, meat and oils. These findings indicate that although some children maintain a relatively stable dietary behaviour during pubertal maturation, others might notably alter their intakes. Nevertheless, only one of the included studies attempted to identify possible determinants of dietary changes during this time period, where, family income, urban-rural residence and mother education were found to be potential predictors of meat, vegetable, fruit and oil intake changes over 6 years [11]. A review on determinants of fruit and vegetable intakes in children and adolescents reported consistent positive associations with family income, parental education, parental intake and home accessibility; a negative association with age; and higher intakes in girls than in boys. However, most of the included studies were based on cross-sectional data and the authors recognised the need for longitudinal analyses [12]. A 2012 longitudinal study testing the association between parental education and intakes of fruit, vegetables, snacks, soft drinks and squash over 20 months, reported that increases in sugar-sweetened beverages were more likely in children with low parental education [13]. Gebremariam et al. assessed the associations of sedentary behaviour on changing intakes of fruits, vegetables, soft drinks, sugar and snacks, and found evidence that high screen-time sedentary behaviour was longitudinally associated with increased consumption of soft drinks and sweets and lower intakes of vegetables [14]. Early onset of puberty was associated with the development of unhealthy lifestyles, such as lower rates of breakfast routines, in a study assessing longitudinal effects of pubertal timing on health behaviours [15]. Additionally, a study in low income adolescents, observed that overweight adolescents were more likely to reduce their energy, fibre and snack food intakes over time [16].

The currently available longitudinal studies suggest that socio-economic environment as well as individual characteristics and behaviours, play an important role in determining food intake changes throughout pubertal maturation. Nevertheless, the available literature is scarce and knowledge in this area is still limited. The need for longitudinal studies assessing differences in dietary behaviours of subjects of both sexes and from different segments of the population has been suggested [12, 17]. To our knowledge, no longitudinal cohort study has yet provided a comprehensive description of habitual dietary intake before and after puberty, assessing both environmental and personal factors as potential determinants of observed changes. Our study aim was hence to examine overall changes in intakes of 17 different food groups representative of total dietary intake, as well as macronutrients and antioxidant vitamins, during this time period; to evaluate the stability of individuals’ intakes over time, and to determine whether specific changes in diet can be predicted by parental education, family income, child education, BMI, pubertal onset and screen-time sedentary behaviour.

## Methods

### Study participants

The present analysis was based on data collected at the 10- and 15-year follow-ups of the ongoing German birth cohort study GINIplus (*G*erman *I*nfant *N*utritional *I*ntervention *plus* environmental and genetic influences on allergy development). Details on the GINIplus study design, recruitment and exclusion criteria have been described previously and can be found elsewhere [18]. In short, healthy full-term new-borns (*n* = 5991) were recruited from obstetric clinics in two different regions of Germany (Munich and Wesel). Infants were allocated to the study intervention arm (randomized to one of three hydrolysed formulae or to conventional cow’s milk) or to the non-intervention arm. Data on health outcomes and covariates were collected by means of identical questionnaires, completed by parents of all children at various time-points. Information on the relevant exposure variables and covariates is given below. To aid reporting of results, the 10-year time-point is hence forth referred to as baseline, and the 15-year time-point as follow-up.

This study was conducted according to the guidelines laid down in the Declaration of Helsinki and all procedures involving human subjects were approved by the local ethics committees (Bavarian Board of Physicians, Board of Physicians of North-Rhine-Westphalia). Written informed consent was obtained from all subjects.

### Dietary intake

Dietary assessment at baseline and follow-up was carried out using a self-administered FFQ, designed and validated to measure 10-year-old children’s usual food and nutrient intake over the past year, and more specifically to estimate energy, fatty acid and antioxidant intake [19]. Due to the uncertain quality of dietary information collected from young children, the FFQ at baseline was addressed to the parents, who completed it along with their children. This was done in order to maximise accuracy by obtaining mutual impact from both the child and the parent [19]. At follow-up, the FFQ was addressed directly to the participants, who were asked to complete it themselves with support of whoever cooked at home, if needed. The FFQ comprised of eighty food items accompanied by several questions about preferred fat and energy contents, preparation methods, diets and food preferences, buying habits and dietary supplement use. To estimate how often food was consumed over the previous year, subjects could choose one of nine frequency categories, including ‘never’, ‘once a month’, ‘2-3 times a month’, ‘once a week’, ‘2-3 times a week’, ‘4-6 times a week’, ‘once a day’, ‘2-3 times a day’ and ‘four times a day or more’. In addition, common portion sizes were assigned for each food item to enable an estimation of quantities. For food items that are difficult to describe in common household measures, coloured photographs from the EPIC (European Prospective Investigation into Cancer and Nutrition) study showing three different portion sizes were included [20]. The 80 FFQ food items were allocated into 41 groups and combined to form 17 major food groups. The categorization systems of a number of sources were compared [21–26] and adapted to the food items present in the FFQ. A list of the resulting food groups is displayed in Table 1. Further details on the development of the FFQ, including food item selection, dietary vitamins, supplement use, and validation methods, have been previously described [19, 27].

**Table 1:** Food groups and list of corresponding food items

Major food group	Food groups	FFQ Food items
1. Fruit	Whole fruit	Apples, Pears
		Tropical fruits
	Berries	Berries
2. Vegetables (excl. potatoes)	Green Leafy	Spinach, chard
		Cruciferous vegetables
		Lettuce
	Red/Orange	Carrots
		Peppers
3. Starchy vegetables	Potatoes	Boiled-, jacket-potato
	Fried potatoes	Chips, croquettes
4. Whole grains	Wholegrain bread	Wholegrain bread/toast
	Wholegrain cereals	Muesli, cereals
5. Refined grains	White breads	White bread/toast
		Bread roll, Pretzel
	Sweet breads	Raisin bread
		Croissant, chocolate bread
	Brown bread	Brown-, rye-, multi-grain
	Refined cereals	Cornflakes
	Pasta	Pasta, noodles
	Rice	Rice
	Pizza	Pizza
	Salty snacks	Snack mixes
6. Meat	Red meat	Pork
		Beef, veal
	Offal	Offal
	Processed meat	Salami
		Leberwurst
		Cold meat
		Bratwurst
		Sausage, Wiener-, pork-sausage
	Poultry	Poultry meat
	Ready-to-eat meals	Ready-to-eat meals with meat
7. Fish	Fresh fish	Freshwater fish
		Salt-water fish
	Canned fish	Bismarck herring, matie
		Canned fish
	Breaded fish	Fish fingers
8. Egg	Egg	Eggs, scrambled/fried
9. Nuts, seeds	Nuts	Nuts
	Seeds	Pumpkin-, pine, sunflower-seed
10. Butter	Butter	Butter
		Butter (in cooking)
11. Margarine	Margarine	Margarine, sunflower spread
		Margarine (in cooking)
	Low-fat margarine	Low-fat margarine
		Low-fat margarine (in cooking)
12. Oils	High MUFA oils	Olive oil
	High PUFA oils	Safflower oil
		Sunflower oil
		Maize germ oil
		Walnut oil
		Vegetable oil
13. Dairy	Milk and milk products	Milk
		Cream cheese, quark (curd)
		Buttermilk, whey
		Hard cheese
		Soft cheese
		Cream, crème fraiche
		Yoghurt, fruit yoghurt
14. Sugar-sweetened foods	Cakes and biscuits	Cream tart
		Pastries
		Biscuits, cookies
		Sponge cake
		Pie
	Chocolate	Chocolate
		Chocolate bars
	Sweets and sugars	Choco-hazelnut spread
		Sugar beet molasses
		Gummy bears
	Dairy products with added sugars	Cocoa, milkshake
		Semolina pudding, rice pudding
		Ice cream
15. Caloric drinks	Sugar-sweetened-drinks	Lemonade, coke, ice tea
		Sport-, energy-drinks
	Fruit and vegetable juices	Squash, fruit nectar
		Fruit juice
		Vegetable juice
		Diluted juice
16. Water	Water	Mineral-, tap water
17. Tea	Tea	Tea

**Fig. 1:**
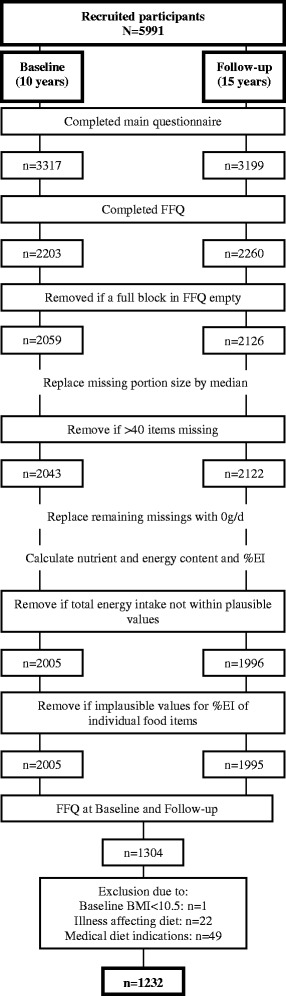
Study population and quality control procedure

A quality control procedure was developed and applied to the FFQ data at both time-points (Fig. 1). This was done based on recommendations by Willett et al. for data cleaning in nutritional epidemiology [28]. Subjects were excluded if a complete block of food items, presented together under the same subheading, was empty (144 at baseline and 134 at follow-up). For each food item, if the intake frequency was provided, but portion size was missing, portion size was replaced by the median obtained from the remaining sex-specific populations. Subjects were excluded if responses to more than 40 food items (50 % of the FFQ) were missing (16 at baseline and 4 at follow-up). Intake frequencies and amounts were then combined to calculate average consumption in grams per day (g/d). Evidence suggests that the presence of intermittent blanks in an otherwise carefully completed FFQ, are best considered as no consumption of the missing food item [28]. Therefore, any remaining missing information on frequency of intake was regarded as “never”, and intake of the specific food item was defined as 0 g/d. Based on the German Food Code and Nutrient Database (BLS) version II.3.1 [29], the corresponding energy and nutrient content per daily grams of intake were calculated for each food item. Total daily energy and nutrient intake was obtained by the sum of daily energy and nutrients of all food items respectively. Intakes relative to total daily energy intake were calculated as the ratio of energy from each food item or macronutrient to the total daily energy intake, and multiplied by 100 to obtain percentage contributions towards total energy intake (%EI). Due to the lack of energy content of water and tea, these food groups were presented in g/day. Furthermore, vitamin intakes were presented in mg/day. Subjects were excluded if total daily energy intake was outside 500-3500 kcal or 800-4000 kcal for females and males respectively (38 subjects at baseline and 126 at follow-up), ranges suggested by Willett et al. in order to avoid substantial loss to follow-up [28]. Further exclusions were made if provided values for %EI of specific food items were implausible (1 subject at follow-up due to extreme rice values: 57 % of total daily energy intake from rice or 620 g/d). Only participants who completed the FFQ at both time-points were included (*n* = 1304). After excluding participants presenting extreme values for co-variables (1 subject), or reporting an illness affecting diet (22 subjects) or medical dietary indications (49 subjects), 1232 participants remained for inclusion in the analyses. Due to the extensive quality control applied at both time-points, the FFQ data in the present study differs from that in previously published papers using only the GINIplus 10-year follow-up dietary data [19, 27].

### Socio-economic environment

#### Parental education and family income

Parental education and family income were used as proxies for socio-economic status (SES). Parental education was defined by the highest level achieved by either the mother or the father, according to the German education system. Children were grouped by low (10 years of education or less) or high (more than 10 years of education) parental education. Family income was categorized by tertiles (low, medium and high), assigned separately and then merged, for the two study centres due to differences in salaries and living costs.

### Individual characteristics and behaviours

#### BMI, pubertal onset, child education level, and screen-time

The focus of the present study was on identifying factors present at childhood, associated with the development of dietary behaviours, and hence only exposure variables measured at baseline were required for the analyses. BMI [kg/m^2^] at baseline was used as a continuous variable, calculated from parental-reported weight and height measurements obtained from the 10-year follow-up questionnaire. Data on pubertal onset (yes/no) were obtained from the 10-year questionnaire, defined as “yes” if parents stated the presence of any of the following: acne or spots, pubic or axillary hair, breast development, menstruation, penis or testicle enlargement, or any other signs of pubertal onset. Data on pubertal stage at follow-up was obtained from a self-rating pubertal development scale [30], and children were categorised into “pre-”, “early-”, “mid-”, “late-” and “post-” pubertal. As the study focus is on changes during puberty, pubertal stage at follow-up was presented for reference, but it must be kept in mind that it is not analogous to the 10-year variable, and hence not comparable. Child education level was defined by the highest level achievable in the secondary school type they attended according to the German education system. Children were grouped analogous to the definition used for parental education, as “low” (schooling programme finalized in 10 years or less) or “high” (schooling programme finalized in more than 10 years). Children who could not be grouped by school type were not included in the analyses. Screen-time was measured at the 10-year follow-up by the amount of time typically spent in front of a screen (television, computer, etc.), reported in 4 categories (ranging from “less than 1 h” to “5 or more”) and categorized as low (≤ 2 h) or high (> 2 h).

### Statistical analysis

To test for differences due to attrition bias, we compared characteristics of participants lost to follow-up (data only at baseline) to those included in the present study analyses, who adhered at follow-up (data at both baseline and follow-up). Categorical variables, presented as percentages, were tested by Fisher's exact test (binary variables) or Pearson’s Chi-squared test (variables with more than 2 levels). Continuous variables, presented as means (standard deviation), were tested by Student’s *t*-test.

The basic characteristics of the study population were described by means (standard deviation) and percentages, separately for females and males. Female and male characteristics were compared using Pearson's Chi-squared Test or Student’s *t*-test for categorical and continuous variables respectively. All further statistical analyses were performed stratified for females and males in order to identify sex-specific differences in dietary behaviours.

#### Average dietary changes

Due to deviation from the normal distribution, food group intake data at baseline and follow-up are presented by the median %EI and 25^th^ and 75^th^ percentiles. Statistically significant differences from baseline to follow-up were tested using the paired Wilcoxon signed rank test.

#### Dietary tracking

Dietary tracking refers to the maintenance of food intake behaviour over time [6]. Each food group was categorized into sex-specific tertiles at baseline and at follow-up: T1 (lowest tertile), T2 (medium tertile) and T3 (highest tertile). Individuals remaining within the same relative tertile of %EI, at baseline and follow-up, were regarded as “tracking” i.e. suggesting stable dietary intakes over time. [11, 16, 31, 32] Tracking coefficients were calculated for each food group by Cohen’s kappa statistic (a measure of agreement between two observations) using linear weights (κ_w_) for kappa values [33]. Coefficients were interpreted based on the following cut-off values as suggested by Landis and Koch [33, 34]: ≤ 0 = poor, 0.01–0.20 = slight, 0.21–0.40 = fair, 0.41–0.60 = moderate, 0.61–0.80 = substantial and 0.81–1 = almost perfect. To test whether individuals tracked significantly within a food group, an exact binomial test was used. Here, the observed percentage of individuals remaining in the same tertile over time (i.e. tracking) was compared to that expected (33.3 %) assuming independence.

#### Associations with dietary changes

In order to avoid false assumptions of linear effects, associations with dietary changes were evaluated categorically using the previously defined tertiles. Possible changes in intakes were identified relative to baseline tertiles: individuals in the lowest tertile T1 at baseline either remained in T1 at follow-up (“tracking in T1”), or increased to tertiles T2 or T3; individuals in the highest baseline tertile T3 either remained in T3 at follow-up (“tracking in T3”), or decreased to T1 or T2. Only individuals in the medium tertile T2 at baseline could either remain in T2 at follow-up (“tracking in T2”), decrease to T1 or increase to T3. Therefore, three regression models were fitted, one for each baseline intake tertile: 1) model for baseline tertile T1 (“increase” vs “tracking in T1”); 2) model for baseline tertile T2 (“increase” and “decrease” vs “tracking in T2”); 3) model for baseline tertile T3 (“decrease” vs “tracking in T3”). The models 1 and 3 were logistic regression models and model 2 was a multinomial logistic regression model. The results are presented as odds ratios with corresponding 95 % confidence interval [OR (95 % CI)]. These regression models tested the associations of dietary changes with parental education level (high vs. low), family income (medium and high vs. low), child education level (high vs. low), pubertal onset at baseline (yes vs. no), baseline BMI, and baseline screen-time (high vs. low). Models were adjusted for possible confounders including age at baseline, baseline energy intake (total daily energy intake [kcal] at 10-year follow-up), diet changes between baseline and follow-up (e.g. starting or stopping a diet in between assessments), study centre (Munich or Wesel), and study intervention arm (assigned to milk formula intervention or control group upon birth). Due to lack of sufficient data in specific cases, certain multinomial regressions were modelled differently: male models for baseline tertile T2 intakes of vegetables, starchy vegetables, refined grain, meat, egg, nuts, butter, margarine and protein, were not adjusted for diet changes; furthermore, the model for T2 starchy vegetable intake in males did not include pubertal onset. For a more thorough interpretation of the regression analyses, we also considered associations between the exposure variables and baseline food intake tertiles, using Pearson’s *χ*^2^ test for categorical variables, and one-way analysis of variance for continuous variables (See Additional file 1: Tables S1a and S1b).

Statistical significance was defined by a two-sided alpha level of 5 %. For the regression analyses we corrected for multiple testing using Bonferroni correction: the alpha level was divided by six, because data were analysed both by sex (two) and baseline intake categories (three) which yields a corrected two sided alpha level of 0.0083 (0.05/(2*3) = 0.0083). All analyses were performed using R version 3.1.0 (https://www.R-project.org/) [35]. Weighted kappa was calculated using the cohen.kappa() function in package “psych” [36], and multinomial regression analysis was performed using the function multinom() in package “nnet” [37].

## Results

In the present analysis 1232 participants (643 females and 589 males) were included with complete FFQ information at both time-points (Fig. 1). Participation at both time-points, compared to participation at baseline only, was higher amongst female subjects, with higher education, subjects with a higher parental education, with medium family income level, with a lower baseline screen-time, or subjects living in Munich (Additional file 2: Table S2).

### Study population

Basic characteristics of the study population stratified by sex are displayed in Table 2. Parental education was mostly high, especially in females (71.4 and 62.9 in females and males respectively). More females (46.4 %) than males (10.9 %) had reached the onset of puberty at baseline, and pubertal development at follow-up was more advanced in females then in males. Mean baseline energy intake was significantly higher in males than females (2105.4 kcal/d (standard deviation = 567.7 kcal/d) in males and 1831.4 kcal/d (488.1 kcal/d) in females), with similar macronutrient proportions in both sexes. Follow-up energy intake was also higher in males, but protein and fat intake was greater in males whereas females consumed more carbohydrates. More females (13.7 %) than males (6.3 %) started or stopped a diet between assessments.

**Table 2:** Basic characteristics of the study population

	Females		Males		*p*-value^a^
	n	% or mean _(SD)_	n	% or mean _(SD)_	
N	643		589		
Parental education level^b^	623		568		
Low (≤ 10 years)	178	28.6	211	37.1	0.002*
High (> 10 years)	445	71.4	357	62.9	
Family income level^c^	592		536		
Low	168	28.4	166	31.0	0.609
Medium	229	38.7	196	36.6	
High	195	32.9	174	32.5	
Child education level	596		552		
Low (≤ 10 years)	210	35.2	215	38.9	0.215
High (> 10 years)	386	64.8	337	61.1	
Pubertal onset at BL	633		579		
Yes	294	46.4	63	10.9	<0.001*
No	339	53.6	516	89.1	
Pubertal onset at FU	553		490		
Pre-pubertal	0	0	6	1.2	<0.001*
Early puberty	0	0	20	4.1	
Mid-puberty	22	4	174	35.5	
Late puberty	450	81.4	286	58.4	
Post-pubertal	81	14.6	4	0.8	
BMI [kg/m^2^]	589	16.7 (2.3)	527	16.8 (2.3)	0.508
Screen-time^d^	631		584		
Low (≤ 2 h)	578	91.6	523	89.6	0.261
High (> 2 h)	53	8.4	61	10.4	
Age at BL [y]	641	11 (0.5)	588	11 (0.5)	0.169
Age at FU [y]	643	15.5 (0.3)	589	15.5 (0.3)	0.961
Energy intake at BL [kcal/day]	643	1831.4 (488.1)	589	2105.4 (562.3)	<0.001*
% Protein at BL	643	14.7	589	14.8	0.597
% Fat at BL	643	30.4	589	31	0.052
% Carbohydrate at BL	643	54.9	589	54.2	0.067
Energy intake at FU [kcal/day]	643	1784.1 (568)	589	2387.4 (657.7)	<0.001*
% Protein at FU	643	14.8	589	15.3	0.001*
% Fat at FU	643	30.1	589	31.3	0.001*
% Carbohydrate at FU	643	55.1	589	53.4	<0.001*
Diet start/stop between BL and FU	630		572		
Yes	86	13.7	36	6.3	<0.001*
No	544	86.3	536	93.7	
Study center	643		589		
Munich	334	51.9	313	53.1	0.717
Wesel	309	48.1	276	46.9	
Study arm	643		589		
Control group	348	54.1	329	55.9	0.579
Infant intervention	295	45.9	260	44.1	

*SD* standard deviation, *BL* baseline, *FU* follow-up

^a^tested by Pearson’s Chi^2^ test (categorical variables) or by Student’s *t*-test; *p-value < 0.05

^b^Highest level achieved by mother or father

^c^Tertiles stratified by study centre and merged

^d^Hours spent on screen-time behaviours.)

#### Average dietary changes

The median (25th percentile; 75th percentile) intakes of food groups (in %EI; in ml/d for tea and water), macronutrients (in %EI), PUFAs (in %EI), and antioxidant vitamins (in mg/d), at baseline and follow-up are presented in Table 3. From baseline to follow-up, females significantly increased their average intakes of vegetables, whole grain, refined grain, oils, tea and water; and decreased their intake of starchy vegetables, meat, margarine, dairy and retinol. However, when excluding females who became vegetarian or vegan (*n* = 25) between baseline and follow-up, the decrease in meat intake was no longer significant. Males significantly increased their average intake of meat, caloric drinks, water, protein, fat, n3 and n6 PUFAs, vitamin C and alpha-tocopherol; and decreased their average fruit, starchy vegetable, margarine, dairy and carbohydrate intakes.

**Table 3:** Changes in average intakes of food groups, macronutrients and vitamins in females and males

	Females				Males			
	Baseline^a^	Follow-up^a^	Change	*p*-value^b^	Baseline^a^	Follow-up^a^	Change	*p*-value^b^
Fruit	4.2 (2.7;6.1)	3.9 (2.3;6.4)		0.568	3.3 (1.9;4.9)	2.2 (1.1;3.8)	(-)	<0.001
Vegetables	1.6 (1.0;2.4)	1.9 (1.1;3.0)	(+)	<0.001	1.2 (0.7;1.8)	1.2 (0.6;1.8)		0.427
Starchy vegetables	2.2 (1.4;3.5)	1.9 (1.2;3.2)	(-)	<0.001	2.1 (1.4;3.3)	1.8 (1.2;2.9)	(-)	<0.001
Whole grains	2.4 (0.7;7.2)	3.0 (0.9;7.6)	(+)	0.026	2.1 (0.3;6.5)	2.4 (0.5;6.0)		0.767
Refined grains	27.8 (23.1;33.9)	28.8 (23.2;35.6)	(+)	0.021	27.4 (21.5;33)	26.7 (21.1;33.3)		0.616
Meat	11.3 (7.7;15.8)	11.1 (6.9;15.4)	(−)	0.043	12.8 (9.3;17.3)	13.7 (10;18.8)	(+)	<0.001
Fish	1.1 (0.6;1.8)	1.1 (0.5;1.8)		0.124	1.3 (0.7;1.9)	1.3 (0.7;2.0)		0.885
Eggs	0.6 (0.3;1.0)	0.6 (0.3;1.0)		0.440	0.5 (0.3;0.9)	0.5 (0.3;1.0)		0.729
Nuts and seeds	0.3 (0.1;0.9)	0.4 (0.0;0.8)		0.940	0.3 (0.1;0.8)	0.3 (0.0;0.9)		0.287
Butter	0.6 (0.1;2.3)	0.7 (0.1;2.4)		0.209	0.6 (0.0;2.3)	0.8 (0.1;2.3)		0.380
Margarine	0.3 (0.0;1.3)	0.2 (0.0;1.1)	(−)	0.013	0.3 (0.0;1.3)	0.2 (0.0;0.9)	(−)	<0.001
Oils	1.2 (0.6;2.4)	1.4 (0.6;2.6)	(+)	0.023	1.1 (0.5;2.3)	1.2 (0.6;2.1)		0.863
Dairy	10.4 (6.8;15.0)	9.2 (5.6;13.6)	(−)	<0.001	10.8 (6.6;16.7)	9.1 (5.5;14.2)	(−)	<0.001
Sugar-sweetened foods	15.7 (9.9;21.8)	15.1 (9.5;21.5)		0.611	15.4 (10.3;22.2)	15.6 (10.3;21.7)		0.996
Caloric drinks	7.1 (2.9;13.0)	6.1 (2.5;12.8)		0.819	8.0 (3.4;14.5)	10.5 (4.5;16.8)	(+)	<0.001
Tea [ml/d]	21.1 (2;89.5)	25.8 (4.4;133.6)	(+)	<0.001	10.1 (0.0;60)	10.2 (0.0;50.8)		0.612
Water [ml/d]	651.0 (339.6;939.1)	906.7 (575.4;1355)	(+)	<0.001	634.4 (277.8;1046)	944.9 (376.3; 1530)	(+)	<0.001
Protein	14.5 (13.0;16.2)	14.6 (12.8;16.2)		0.229	14.8 (13.2;16.3)	15.2 (13.4;17.0)	(+)	<0.001
Fat	29.8 (26.2;34.0)	29.7 (26.5;33.6)		0.195	30.5 (27.5;34.3)	30.8 (27.4;35.3)	(+)	<0.001
Carbohydrate	55.4 (50.9;59.5)	55.5 (50.9;59.5)		0.062	54.8 (49.8;58.8)	53.5 (48.5;58.3)	(−)	<0.001
n3 PUFA	0.6 (0.5;0.6)	0.6 (0.5;0.7)		0.721	0.5 (0.5;0.6)	0.6 (0.5;0.7)	(+)	<0.001
n6 PUFA	3.8 (3.3;4.5)	3.9 (3.3;4.7)		0.818	3.9 (3.3;4.6)	4.0 (3.3;4.7)	(+)	<0.001
Retinol [mg/d]	0.4 (0.3;0.5)	0.3 (0.2;0.5)	(−)	<0.001	0.4 (0.3;0.7)	0.5 (0.3;0.7)		0.053
Beta Carotene [mg/d]	4.0 (2.6;5.9)	3.9 (2.4;5.8)		0.752	3.5 (2.2;5.4)	3.3 (2.0;5.2)		0.076
Vitamin C [mg/d]	99.4 (71.3;136.8)	97.7 (69.0;146.1)		0.264	98 (68.3;130.7)	102.2 (72.4;140.9)	(+)	0.019
alpha tocopherol [mg/d]	7.8 (6.1;9.8)	7.9 (6.0;10.4)		0.130	8.2 (6.4;10.4)	9.0 (7.1;11.5)	(+)	<0.001

^a^Median (25^th^ percentile; 75^th^ percentile), presented in %EI unless stated otherwise

^b^Paired Wilcoxon rank sum test; (+) = significant increase from baseline to follow-up: *p*-value < 0.05; (−) = significant decrease from baseline to follow-up: *p*-value < 0.05

#### Dietary tracking

Tracking coefficients and percentages of individuals tracking are shown for females and males in Table 4. Based on the kappa coefficients, both sexes presented fair tracking for most food groups, macronutrients, PUFAs and vitamins (κ = 0.21-0.4). Exceptions in both sexes were butter, margarine and tea, which showed moderate tracking levels (κ = 0.41-0.6). Furthermore, oil, fat, carbohydrates and retinol, in females, and alpha-tocopherol in males showed only slight tracking levels (κ = 0.01-0.20). Both females and males tracked significantly for all food groups, macronutrients, PUFAs and vitamins (i.e. significantly more subjects remained in the same relative tertile from baseline to follow-up than expected by chance).

**Table 4:** Tracking coefficients and percentage of individuals tracking in females and males

	Females		Males	
	Coefficient (κ_w_)^a^	%^b^	Coefficient (κ_w_)^a^	%^b^
Expected^c^		33.3		33.3
Fruit	0.259 (0.20;0.32)	45.9	0.389 (0.33;0.45)	54.2
Vegetables	0.311 (0.25;0.37)	49.8	0.309 (0.25;0.37)	47.7
Starchy vegetables	0.371 (0.31;0.43)	52.1	0.313 (0.25;0.37)	48.9
Whole grains	0.245 (0.18;0.31)	46.8	0.263 (0.20;0.33)	46.2
Refined grains	0.238 (0.18;0.30)	44.8	0.221 (0.16;0.28)	44.0
Meat	0.273 (0.21;0.33)	46.3	0.259 (0.20;0.32)	46.0
Fish	0.287 (0.23;0.35)	47.3	0.286 (0.22;0.35)	46.3
Egg	0.224 (0.16;0.28)	44.0	0.259 (0.20;0.32)	46.9
Nuts and seeds	0.217 (0.16;0.28)	43.1	0.298 (0.23;0.36)	48.7
Butter	0.451 (0.40;0.51)	57.9	0.481 (0.42;0.54)	60.3
Margarine	0.469 (0.41;0.52)	59.3	0.455 (0.40;0.51)	58.6
Oils	0.185 (0.12;0.25)	42.8	0.263 (0.20;0.33)	47.5
Dairy	0.252 (0.19;0.31)	46.3	0.286 (0.22;0.35)	48.0
Sugar sweetened foods	0.259 (0.20;0.32)	47.0	0.240 (0.18;0.30)	46.2
Caloric drinks	0.315 (0.25;0.37)	50.1	0.389 (0.33;0.45)	53.8
Tea	0.428 (0.37;0.48)	56.8	0.432 (0.37;0.49)	56.0
Water	0.311 (0.25;0.37)	48.5	0.391 (0.33;0.45)	54.0
Protein	0.220 (0.16;0.28)	43.2	0.259 (0.20;0.32)	46.0
Fat	0.196 (0.14;0.26)	41.4	0.225 (0.16;0.29)	44.3
Carbohydrate	0.189 (0.13;0.25)	40.6	0.240 (0.18;0.30)	45.2
n3 PUFA	0.238 (0.18;0.30)	45.7	0.217 (0.15;0.28)	43.6
n6 PUFA	0.224 (0.16;0.28)	44.6	0.240 (0.18;0.30)	45.5
Retinol [mg/d]	0.196 (0.13;0.26)	44.3	0.313 (0.25;0.37)	49.1
Beta Carotene [mg/d]	0.304 (0.24;0.36)	49.5	0.332 (0.27;0.39)	49.9
Vitamin C [mg/d]	0.259 (0.20;0.32)	47.1	0.202 (0.14;0.27)	43.6
alpha tocopherol [mg/d]	0.206 (0.15;0.27)	42.8	0.126 (0.06;0.19)	38.0

^a^Tracking coefficient of weighted Cohen’s Kappa (95 % CI)

^b^Individuals (%) remaining in the same relative tertile from baseline to follow-up

^c^Expected (%) individuals remaining in the same tertile assuming unity

#### Associations with dietary changes

Dietary changes presenting significant associations (change vs. tracking) with any of parental education level, family income, child education level, pubertal onset, BMI and screen-time are shown in Tables 5 and 6 for females and males respectively. Results for the regression analyses on the remaining food groups, macronutrients, PUFAs, or vitamins are presented in Additional file 3: Table S3.

**Table 5:** Associations^a^ with dietary intake changes stratified by baseline intake tertile in females

Reference	Tracking in T1^b^	Tracking in T2^c^		Tracking in T3^d^
Change	Increase	Increase	Decrease	Decrease
Whole grains				
ParEdu high	1.8 (0.7;4.2)	1.1 (0.4;3.1)	0.6 (0.2;1.6)	0.7 (0.3;1.9)
Income med	1.2 (0.5;2.8)	0.3 (0.1;0.8)	0.2 (0.1;0.7)*	0.4 (0.2;1.1)
Income high	0.6 (0.2;1.7)	0.1 (0.0;0.5)*	0.3 (0.1;1.0)	1.2 (0.4;3.1)
ChildEdu high	0.9 (0.4;2.0)	1.7 (0.6;4.7)	1.7 (0.6;4.4)	0.6 (0.2;1.3)
Puberty yes	0.9 (0.4;1.8)	0.9 (0.4;2.1)	1.1 (0.5;2.5)	0.8 (0.4;1.6)
BMI	0.9 (0.8;1.1)	1.0 (0.8;1.2)	1.1 (0.9;1.3)	1.1 (0.9;1.3)
Screen high	0.3 (0.1;1.1)	0.2 (0.0;2.2)	2.2 (0.6;7.7)	3.0 (0.5;16.9)
Nuts				
ParEdu high	3.8 (1.7;8.8)*	1.8 (0.6;5.4)	0.8 (0.3;2.1)	0.6 (0.2;1.4)
Income med	0.5 (0.2;1.1)	1.9 (0.7;5.2)	3.2 (1.0;9.8)	1.8 (0.7;4.4)
Income high	0.4 (0.1;1.1)	0.6 (0.2;1.8)	2.1 (0.6;6.7)	1.4 (0.5;3.8)
ChildEdu high	0.8 (0.4;1.7)	0.8 (0.3;2.0)	1.1 (0.4;3.0)	1.4 (0.6;3.1)
Puberty yes	1.1 (0.6;2.3)	1.1 (0.5;2.6)	1.3 (0.6;3.1)	0.5 (0.3;1.0)
BMI	0.9 (0.8;1.1)	1.0 (0.9;1.3)	0.9 (0.8;1.1)	0.9 (0.8;1.1)
Screen high	0.4 (0.1;1.2)	1.4 (0.3;7.3)	2.2 (0.5;10.4)	0.5 (0.1;2.1)
Protein				
ParEdu High	1.5 (0.7;3.4)	1.0 (0.4;2.7)	1.1 (0.4;2.9)	0.3 (0.1;0.9)
Income med	0.9 (0.4;2.1)	0.4 (0.1;1.1)	0.7 (0.2;2.1)	0.6 (0.2;1.6)
Income high	1.3 (0.6;3.3)	0.9 (0.3;2.7)	1.8 (0.6;5.9)	0.6 (0.2;2.1)
ChildEdu high	0.6 (0.3;1.3)	0.7 (0.3;1.8)	0.5 (0.2;1.2)	0.9 (0.4;2.2)
Puberty yes	1.0 (0.5;2.1)	1.4 (0.6;3.2)	1.1 (0.5;2.5)	1.1 (0.5;2.3)
BMI	1.0 (0.8;1.2)	1.0 (0.9;1.2)	1.0 (0.8;1.2)	0.7 (0.6;0.9)*
Sed high	0.8 (0.3;2.2)	1.0 (0.2;4.5)	0.2 (0.0;2.0)	2.5 (0.7;9.1)
Retinol				
ParEdu High	0.7 (0.3;1.7)	1.2 (0.5;3.1)	1.3 (0.5;3.7)	1.1 (0.5;2.4)
Income med	0.8 (0.3;2.0)	0.6 (0.2;1.8)	0.2 (0.1;0.6)*	0.6 (0.2;1.4)
Income high	1.2 (0.5;3.4)	0.7 (0.2;2.4)	0.2 (0.1;0.7)	0.8 (0.3;2.1)
ChildEdu high	0.9 (0.4;2.2)	1.1 (0.4;2.8)	0.3 (0.1;0.8)	0.6 (0.3;1.4)
Puberty yes	2.1 (1.0;4.3)	0.7 (0.3;1.7)	0.3 (0.1;0.9)	0.9 (0.5;1.9)
BMI	1.0 (0.9;1.2)	1.0 (0.8;1.2)	1.0 (0.8;1.2)	1.2 (1.0;1.4)
Sed high	0.6 (0.2;1.9)	0.8 (0.2;2.8)	0.2 (0.0;1.0)	0.2 (0.0;1.1)
Vitamin C				
ParEdu High	1.0 (0.4;2.3)	0.6 (0.2;1.7)	0.2 (0.1;0.5)*	1.4 (0.6;3.3)
Income med	0.5 (0.2;1.1)	1.5 (0.5;4.5)	1.0 (0.3;2.9)	0.3 (0.1;0.8)
Income high	0.4 (0.2;1.1)	3.2 (0.9;10.6)	1.3 (0.4;4.6)	0.2 (0.1;0.6)*
ChildEdu high	1.5 (0.7;3.3)	0.6 (0.3;1.5)	0.8 (0.3;2.0)	2.2 (0.9;5.2)
Puberty yes	1.5 (0.7;3.2)	0.4 (0.2;0.9)	0.7 (0.3;1.9)	1.3 (0.6;2.5)
BMI	1.0 (0.8;1.1)	1.2 (1.0;1.5)	1.2 (1.0;1.4)	1.1 (1.0;1.3)
Sed high	0.8 (0.3;2.3)	0.9 (0.2;5.0)	1.2 (0.2;6.5)	1.7 (0.5;6.2)

ParEdu high: parental education (high vs. low); Income med/high: family income (medium/high vs. low); ChildEdu high: child education (high vs. low); Puberty yes: pubertal onset at baseline (yes vs. no); Screen high: screen-time at baseline (high vs. low).

*p-value < 0.0083 (Bonferroni correction for multiple testing: 0.05/6)

^a^Odds ratio (95 % CI)

^b^Logistic regression (increase vs. tracking in lowest tertile)

^c^Multinomial logistic regression (increase or decrease vs. tracking in medium tertile)

^d^Logsitic regression (decrease vs. tracking in highest tertile)

**Table 6:** Associations^a^ with dietary intake changes stratified by baseline intake tertile in males

Reference	Tracking in T1^b^	Tracking in T2^c^		Tracking in T3^d^
Change	Increase	Increase	Decrease	Decrease
Egg^e^				
ParEdu high	0.2 (0.1;0.5)*	0.6 (0.2;1.9)	0.7 (0.3;2.1)	0.7 (0.3;1.7)
Income med	2.2 (0.8;6.0)	1.2 (0.4;3.6)	1.0 (0.4;3.0)	1.2 (0.5;3.0)
Income high	2.1 (0.7;6.4)	0.4 (0.1;1.5)	0.8 (0.3;2.4)	1.6 (0.6;4.1)
ChildEdu high	1.0 (0.4;2.5)	3.0 (1.0;9.2)	1.2 (0.4;3.1)	1.4 (0.6;3.4)
Puberty yes	2.3 (0.6;8.7)	7.3 (1.3;39.9)	3.5 (0.6;19.9)	1.8 (0.6;5.1)
BMI	0.9 (0.8;1.1)	1.0 (0.8;1.2)	0.9 (0.8;1.1)	1.0 (0.9;1.1)
SedBeh high	0.5 (0.2;1.3)	1.3 (0.3;5.3)	0.7 (0.1;3.3)	1.4 (0.4;4.4)
Margarine^e^				
ParEdu high	0.5 (0.2;1.5)	0.9 (0.3;2.8)	3.8 (0.9;15.2)	1.0 (0.4;2.5)
Income med	1.5 (0.5;4.6)	0.5 (0.2;1.5)	0.9 (0.3;3.3)	1.3 (0.5;3.4)
Income high	1.3 (0.4;3.8)	0.7 (0.2;2.3)	0.7 (0.2;2.9)	3.6 (1.1;11.5)
ChildEdu high	0.8 (0.3;2.3)	0.4 (0.1;1.2)	0.2 (0.1;0.9)	0.6 (0.2;1.3)
Puberty yes	0.9 (0.2;3.2)	1.5 (0.4;6.3)	2.1 (0.5;9.4)	1.6 (0.4;6.1)
BMI	1.3 (1.1;1.6)*	0.8 (0.7;1.0)	0.9 (0.8;1.2)	1.0 (0.9;1.2)
SedBeh high	1.5 (0.4;6.4)	0.9 (0.3;3.4)	1.2 (0.3;5.0)	2.8 (0.9;8.8)
Sugar-sweetened foods				
ParEdu high	1.9 (0.7;5.3)	0.6 (0.2;1.9)	2.5 (0.6;10.7)	1.1 (0.4;2.7)
Income med	1.6 (0.5;4.5)	3.0 (0.9;10.0)	5.5 (1.4;22.5)	1.2 (0.5;2.9)
Income high	2.4 (0.7;7.5)	0.9 (0.3;3.0)	0.8 (0.2;3.3)	0.8 (0.3;2.3)
ChildEdu high	0.1 (0.1;0.4)*	2.5 (0.8;7.6)	2.4 (0.6;8.9)	0.7 (0.3;1.6)
Puberty yes	1.3 (0.4;3.8)	0.3 (0.0;1.7)	0.5 (0.1;3.5)	11.3 (1.3;98.4)
BMI	0.9 (0.7;1.0)	0.8 (0.6;1.0)	1.0 (0.8;1.3)	0.9 (0.8;1.1)
SedBeh high	2.1 (0.5;8.6)	2.1 (0.5;9.0)	4.0 (0.7;23.9)	1.0 (0.4;2.8)
n3 PUFA				
ParEdu high	0.2 (0.1;0.5)*	0.5 (0.1;1.6)	1.0 (0.3;3.4)	1.0 (0.4;2.4)
Income med	1.4 (0.5;3.9)	1.5 (0.4;5.1)	0.9 (0.3;3.0)	1.4 (0.6;3.4)
Income high	1.6 (0.6;4.4)	1.4 (0.4;5.0)	1.4 (0.4;4.8)	1.5 (0.6;4.1)
ChildEdu high	0.6 (0.2;1.7)	0.9 (0.3;2.5)	0.8 (0.3;2.1)	1.1 (0.5;2.5)
Puberty yes	1.2 (0.3;4.5)	1.0 (0.3;4.0)	0.5 (0.1;2.1)	0.8 (0.3;2.7)
BMI	0.7 (0.6;0.9)*	0.8 (0.7;1.0)	1.0 (0.8;1.2)	1.1 (0.9;1.3)
SedBeh high	0.3 (0.1;0.9)	1.3 (0.3;5.7)	0.6 (0.1;3.0)	1.1 (0.3;3.8)
Vitamin C				
ParEdu high	0.6 (0.2;1.5)	1.0 (0.3;3.1)	0.7 (0.2;2.0)	2.1 (0.8;5.5)
Income med	1.1 (0.5;2.8)	0.7 (0.2;2.3)	0.5 (0.2;1.5)	1.5 (0.6;4.2)
Income high	1.3 (0.5;3.7)	0.7 (0.2;2.6)	0.7 (0.2;2.3)	1.5 (0.5;4.0)
ChildEdu high	1.6 (0.7;4.1)	1.3 (0.5;3.5)	1.4 (0.5;3.7)	1.2 (0.5;3.2)
Puberty yes	1.2 (0.3;4.1)	0.7 (0.1;4.2)	1.6 (0.4;6.9)	1.7 (0.5;5.6)
BMI	1.3 (1.1;1.6)*	1.0 (0.8;1.2)	1.0 (0.8;1.2)	0.9 (0.8;1.1)
SedBeh high	0.5 (0.2;1.5)	1.6 (0.5;5.2)	0.8 (0.2;3.0)	4.2 (0.9;19.3)

ParEdu high: parental education (high vs. low); Income med/high: family income (medium/high vs. low); ChildEdu high: child education (high vs. low); Puberty yes: pubertal onset at baseline (yes vs. no); Screen high: screen-time at baseline (high vs. low)

*p-value < 0.0083 (Bonferroni correction for multiple testing: 0.05/6)

^a^Odds ratio (95 % CI)

^b^Logistic regression (increase vs. tracking in lowest tertile)

^c^Multinomial logistic regression (increase or decrease vs. tracking in medium tertile)

^d^Logsitic regression (decrease vs. tracking in highest tertile)

^e^Multinomial regression not adjusted for diet change

Females with higher compared to lower parental education level, and with low (T1) baseline nut intakes, were more likely to increase nut intake over time [OR = 3.8; 95 % CI = (1.7, 8.8)]. Similarly, high parental education females were less likely to reduce medium (T2) vitamin C intakes [0.2 (0.1, 0.5)]. Females with medium (T2) baseline whole grain intakes and medium family income, were less likely to reduce their intakes [0.2 (0.1, 0.7)] than females with a low family income; whereas those with high family income were less likely to increase their whole grain intakes [0.1 (0.0, 0.5)]. Females with medium family income and medium (T2) baseline retinol intake were less likely to decrease their intakes [0.2 (0.1, 0.6)]. Furthermore, high family income level females with high (T3) vitamin C intakes were less likely to reduce their intakes over time [0.2 (0.1, 0.6)]. Finally, BMI in females was negatively associated with decreasing high (T3) protein intakes [0.7 (0.6, 0.9)], i.e. higher BMI females were more likely to maintain high protein intakes at follow-up than to reduce them.

Compared to low parental education, males with high parental education, and low (T1) baseline egg intakes, were less likely to increase their egg consumption [0.2 (0.1, 0.5)]. Similarly, those with low n3 PUFA intakes were less likely to increase their intakes [0.2 (0.1, 0.5)]. Children with high education level and low (T1) baseline sugar-sweetened food intakes were less likely to increase their intakes [0.1 (0.1, 0.4)]. BMI in males was positively associated with increased margarine [1.3 (1.1, 1.6)] and vitamin C intakes [1.3 (1.1, 1.6)], when baseline intakes were low (T1); whilst a negative association was seen with increasing n3 PUFA [0.7 (0.6, 0.9)], i.e. higher BMI males were more likely to increase low baseline margarine and vitamin C intakes, and to maintain low n3 PUFA intakes at follow-up.

## Discussion

In the present study we evaluated changes in intakes of 17 food groups, as well as macronutrients, and antioxidant vitamins, using repeated FFQ data from the 10- and 15-year follow-up assessments of the German GINIplus birth cohort study. We observed overall dietary intake changes occurring within the study population, evaluated individual levels of dietary stability (tracking), and identified socio-economic factors, and individual characteristics and behaviours which may be associated with specific dietary changes during the transition from childhood to adolescence.

The few studies available describing habitual dietary intake during puberty, differ in terms of study design, follow-up period, data collection methods, age of subjects, and study location [11, 13, 31, 32, 38, 39]. Dietary behaviours observed, range from specific food items or food groups [13, 31, 38–40] to broader dietary patterns, including a range of foods [11, 16, 41]. Comparison with other studies is hence limited, especially because available longitudinal studies are scarce; however, despite these differences, some similarities and inconsistencies between our and previous study findings, are worth mentioning.

### Average changes in dietary intake

Average intakes of food groups changed significantly in both males and females. Both sexes presented a decrease in intakes of starchy vegetables, dairy and margarine, and an increase in total water intake. Meat intake increased in males and decreased in females (mainly due to subjects changing towards a vegetarian or vegan diet). Males also reduced fruit intake and increased caloric drinks, while females increased intakes of whole and refined grains, vegetables, oils and tea. As in our study, a study in Swedish adolescents aged 15 at baseline, and followed up at ages 17 and 21, reported that changes in food group intakes in males were less frequent than in females, suggesting a greater tendency in females to modify their diet during pubertal maturation and throughout adolescence [38]. Nevertheless, these changes did not seem to impact the overall intakes of macronutrients and vitamins in females, who presented only decreased retinol intakes. As meat and dairy are sources of this vitamin [42], the reduced consumption of these food groups in females might explain the lower retinol intakes. Males however, significantly increased protein and fat intakes, as well as n3 and n6 PUFAs, vitamin C and alpha-tocopherol, and decreased carbohydrates. Furthermore, food groups presenting changes in the previously mentioned study were similar to those in our study: females decreased fat spread, milk and meat intakes, and increased pasta intake from 15 to 17 years. At 21 years females had further reduced their meat intake and males had reduced fruit intake [38]. An increased consumption of caloric drinks in adolescence has also been observed previously in Norwegian [13] and German populations, especially in males [40].

### Dietary tracking

Dietary tracking assessed the stability of food intakes within the study population. Females and males presented “fair” levels of tracking for all food groups, except for butter, margarine, and tea, which revealed stronger tracking; and oil, fat, carbohydrate and retinol in females and alpha-tocopherol in males, which showed only slight tracking. Previous studies on tracking of dietary behaviour in females and males during puberty have reported similar (slight to moderate) tracking levels for food groups such as fruit and vegetables [13, 16], caloric drinks [13, 31], dairy [31] or meat [11], among others. The present results suggest a possible overlap of dietary behaviours observed in other countries, although this may be limited due to sociocultural differences. We noted that food groups indicating greater stability were also amongst those presenting highly significant changes in average intakes. For example, average margarine intake decreased significantly over time, but margarine also presented the highest tracking coefficients in both females and males. These results are not necessarily contradictory as it is possible for a child to significantly modify his/her intake of a specific food group, while remaining in the same position relative to others in the sample (indicative of tracking). We performed further sensitivity analyses to determine if specifically non-tracking participants were responsible for the observed changes, but this was not the case. These results suggest that in our study sample, average intake changes observed during puberty in food groups such as margarine, starchy vegetables, fruit and caloric drinks, follow sex-specific secular trends, where the “order of the children by intake” remains but the overall median intakes are altered.

### Associations with dietary changes

In the present study, the association of dietary intake changes, with selected indicators of socio-economic status and individual characteristics differed amongst females and males for different food groups, macronutrients and vitamins, and according to baseline intake levels. Studies on the determinants of changes in dietary intake during puberty are limited. Wang et al. [11], reported that children’s dietary intake patterns can be predicted by family income, urban-rural residence, maternal education and baseline dietary intakes. In our study we observed significant associations of dietary intake with parental education, family income, child education and BMI. Given that the consumption of nuts, whole grains, vitamin C and retinol are frequently associated with health-benefits [42–44], our findings suggest that higher SES in females, represented by higher parental education and family income, may promote an increased consumption (nuts) or at least the maintenance of higher intakes (whole grain, vitamin C and retinol) of certain healthier foods and nutrients during puberty. On the other hand, our results also indicate that females with lower family income were more likely to increase whole grain intakes than those with high income. Despite typically being more expensive [45], increasing whole grain products may be an attainable goal in children with less resources making efforts to improve their diet as they grow older. In males, higher parental education was associated with maintenance of low egg and n3 PUFA intakes as opposed to increasing intakes. Egg intake has been previously associated with unhealthy lipid profiles in humans [46]. Adolescent males with higher educated parents may be more informed with regards to dietary advice [47], and eggs may hence be eaten sparingly. Eggs are also a source of n3 PUFA, which may in turn remain low in the same male subgroup of parental education, even though n3 PUFA has been associated with beneficial health effects [48]. Higher child education in males was associated with tracking low intakes of sugar-sweetened foods, rather than increasing them. Those with higher education levels may be more aware of the negative relationship between health and carbohydrate-rich diets, especially sugar [49, 50], and hence attempt to lower their intakes [51].

Higher BMI was associated with tracking of high protein intake in females. In males BMI was positively associated with increasing margarine, and vitamin C, and with maintenance of n3 PUFA levels in the lowest baseline intake tertiles. High BMI is often associated with unhealthy dietary behaviours [52–54], however in the present study BMI does not seem to be a predominant predictor of unhealthy dietary change during adolescence. This could be due to common underreporting of unhealthy foods in overweight subjects [55, 56] (margarine may be regarded as healthy and hence not underreported, given its lower content of saturated fats compared to butter [57]). The lack of associations with BMI could also be explained by possible earlier influences of the exposure variable on food intake at baseline. Dietary behaviours already established before the baseline assessment could indicate an intake threshold was reached before puberty, impeding further change in that direction, e.g. higher BMI was associated with high starchy vegetable, meat, water and protein intakes at baseline (see Additional file 1: Table S1). Similarly, parental education level, child education and screen-time also showed significant differences in intakes of a number of food groups at baseline (e.g. higher parental education associated with higher intakes of grains, butter and oil and lower intakes of starchy vegetables and margarine; higher child education with higher fruit, wholegrain and butter intakes and lower intakes of starchy vegetables, meat and sugar-sweetened foods; and higher screen-time associated with lower fruit, vegetables, wholegrain and beta-carotene in both females and males). However earlier influences must be interpreted with caution, as these were cross-sectional associations and reverse causality cannot be excluded as there is no previous dietary data available for longitudinal analyses before the 10 year assessments. We hence highlight the importance of longitudinal analyses in investigating associations with dietary intake changes.

#### Strengths and limitations

The present study benefits from a large study population of males and females within two distinct German regions. The longitudinal nature of this study, covering a 5-year period from childhood into adolescence, is a key aspect which allows us to add to the limited knowledge regarding dietary behaviour changes during adolescence. The large amount of descriptive data, obtained from the GINIplus cohort, along with comprehensive dietary data from the food frequency questionnaires, provide a thorough overview of habitual dietary intake during two key stages, as well as possible determinants of changes in intakes during pubertal maturation.

Several possible shortcomings of the study must be considered. Even though study sampling was primarily population-based, our study population for analysis is, as in every cohort study, subject to selection bias, and thus the findings cannot be considered as representative for the study area. Owing to non-random loss-to-follow-up, the cohort on which the present analysis is based underrepresents children from lower social classes. The true social inequalities might therefore be even stronger than reported here. This would also explain the relatively few associations with parental education observed in our study despite the literature suggesting otherwise [11, 38].

The large number of food groups assessed, and the possibility that they may be correlated, increases the chance for type 1 error. We tried to account for this by using Bonferroni correction for multiple testing, lowering our two-sided alpha level to 0.0083. Furthermore, thorough analyses of interaction effects between independent variables were not possible. Despite our large sample size, analyses by baseline intake levels and sex already resulted in partly small groups, and hence the data could not provide enough power for further stratification.

The FFQ used in the present study was designed with a special focus on energy, antioxidant and fatty acid intake. Hence, the food item list may underestimate intakes of other food items not included in the questionnaire. The same FFQ was administered at 10 and 15 years in order to use a consistent methodology to measure dietary changes over time. The FFQ was designed for measuring dietary intake in school-aged children, and validated using 24 h-dietary recalls. The test-retest performance of the questionnaire was not assessed, which is a limitation in the present study. Nevertheless, at 10 years it proved applicable and comprehensible, and produced highly plausible dietary estimates, justifying its use in future epidemiological studies [19]. A study testing the use of an FFQ in older children and adolescents aged 9-18 years, found it to be reproducible regardless of age [58]; and a review summarizing the validity and reliability of food frequency questionnaires in children and adolescents, reported mainly strong correlations in studies reporting test-retest reliability [59]. We hence believe that our results should not be majorly affected by this limitation. Furthermore, the FFQ was completed by a parent alongside the participant at baseline, and by the participants themselves with support of whoever cooked at home, at follow-up. It is generally believed that children before the age of 12 have difficulties recalling intakes and understanding portion sizes, and have a more limited knowledge of foods, all of which constrains their ability to self-report without parental assistance [60]. Furthermore, studies have reported that the parental indication of children’s dietary intake appears to be moderately valid [28]. Therefore, a combined effort in the completion of the FFQ at baseline was considered appropriate to maximise response accuracy. Nevertheless, inter-reporter differences cannot be excluded, for example due to varying perceptions of quantification measures, or due to selective under- or over-reporting (in response to perceptions of social desirability). Therefore, the observed results could, to some extent represent reporting error at different time points, rather than actual dietary changes over time.

Finally, the possible role of secular trends shaping dietary intake over time cannot be excluded [61]. Nevertheless, in identifying possible determinants, intakes were categorised by tertiles and hence only changes large enough to produce a tertile shift over time (e.g. T1 to T2 or T3) were classified as changing. Therefore, while small changes which were common across the entire population could have indicated trends, our regression analyses most likely reveal individual associations with greater intake changes. Unfortunately, categorisation of data implies certain loss of information. However, using tertile categories rather than actual intakes, is commonly used to measure tracking [11, 16, 31, 32] and was preferred, in order to overcome the non-normal distribution of the dietary data, as well as possible problems of under- or over-reporting.

## Conclusions

Average dietary intakes changed significantly from childhood to adolescence. Nevertheless a fair degree of tracking was observed, suggesting the presence of general, sex-specific trends in dietary behaviour during this period. Dietary intake changes were most frequently associated with socio-economic environment, where females with high SES tended towards healthier dietary behaviours. Associations with child education and BMI were also observed for some food groups and nutrients, while no effect was seen between intake changes and screen-time or pubertal onset. Our results support the rationale for dietary interventions targeting children in order to positively influence dietary changes during puberty. We suggest that sex-specific subpopulations, such as children with lower SES, or lower education levels, should be considered for further impact. We further highlight the need for longitudinal studies in this topic given its relevance in the development of public health nutrition strategies.

## Additional files

Additional file 1:
**Associations between exposure variables and baseline food intake tertiles** (PDF 628 kb) Additional file 2:
**Comparison of lost-to-follow-up and not-lost-to-follow-up participants** (PDF 264 kb) Additional file 3:
**Associations with dietary intake changes stratified by baseline intake tertile** (PDF 303 kb)

## Abbreviations

BMI: Body mass index
FFQ: Food frequency questionnaire
%EI: Percentage contribution towards total energy intake
SES: Socio-economic status
SD: Standard deviation
OR: Odds ratio
CI: Confidence interval
T1: Lowest intake tertile
T2: Medium intake tertile
T3: Highest intake tertile
