# Supplementary material for: Changes in dietary intake during puberty and their determinants: results from the GINIplus birth cohort study
Source: BMC Public Health. 2015 Sep 2;15:841. doi: 10.1186/s12889-015-2189-0 (PMC4556194; doi:10.1186/s12889-015-2189-0)
Supplement: Additional file 1: — Associations between exposure variables and baseline food intake tertiles (PDF 628 kb) [file 12889_2015_2189_MOESM1_ESM.pdf]

Additional file 1. Associations between exposure variables and baseline food intake tertiles

Supplementary Table 1a Associations between exposure variables and baseline food intake tertiles in females

	Parental educ.[1]		Family income level[1]			School level[1]		Puberty[1]		BMI[2]	Screen time[1]	
	lower	higher	lower	med	higher	lower	higher	yes	no		lower	higher
N	178	445	168	229	195	210	386	294	339	589	578	53
**Fruit**												
T1	39.3	31.2	38.7	34.5	26.2	41.9	30.8	36.7	31.3	17.0 (2.4)	31.1	54.7
T2	31.5	34.4	31.0	32.8	37.4	34.3	31.1	29.9	36.9	16.6 (2.4)	33.9	30.2
T3	29.2	34.4	30.4	32.8	36.4	23.8	38.1	33.3	31.9	16.6 (2.3)	34.9	15.1
p-value	0.147		0.144			0.001*		0.153		0.285	0.001*	
**Vegetable**												
T1	41.0	30.8	33.9	36.2	31.3	38.1	31.1	33.0	33.6	16.7 (2.4)	31.3	52.8
T2	32.6	33.5	35.1	34.9	29.2	34.3	33.4	34.4	33.0	16.9 (2.3)	34.8	22.6
T3	26.4	35.7	31.0	28.8	39.5	27.6	35.5	32.7	33.3	16.6 (2.3)	33.9	24.5
p-value	0.026*		0.199			0.100		0.941		0.644	0.006*	
**Starchy vegetables**												
T1	23.6	37.5	29.8	35.4	36.4	25.2	36.8	34.0	33.0	16.8 (2.4)	34.3	28.3
T2	36.0	31.7	35.7	31.4	31.8	33.8	33.9	35.7	31.3	16.3 (1.8)	33.4	30.2
T3	40.4	30.8	34.5	33.2	31.8	41.0	29.3	30.3	35.7	17.1 (2.7)	32.4	41.5
p-value	0.003*		0.705			0.004*		0.305		0.008*	0.389	
**Wholegrain**												
T1	41.6	30.1	35.7	31.4	31.3	45.2	28.0	27.9	38.1	16.5 (2.4)	32.5	39.6
T2	30.3	35.3	31.5	37.1	33.3	29.5	35.2	36.7	30.7	16.8 (2.5)	33.6	35.8
T3	28.1	34.6	32.7	31.4	35.4	25.2	36.8	35.4	31.3	16.9 (2.2)	33.9	24.5
p-value	0.023*		0.707			<0.001*		0.025*		0.351	0.349	
**Refined grain**												
T1	41.6	29.7	37.5	28.4	33.8	37.6	32.4	33.7	32.4	16.5 (2.3)	32.7	37.7
T2	29.8	35.1	29.8	34.5	35.4	32.9	34.5	32.3	34.5	16.8 (2.4)	33.7	28.3
T3	28.7	35.3	32.7	37.1	30.8	29.5	33.2	34.0	33	16.9 (2.4)	33.6	34.0
p-value	0.017*		0.301			0.416		0.842		0.214	0.669	
**Meat**												
T1	26.4	35.7	29.2	35.4	33.8	27.6	35.5	31.6	34.8	16.4 (1.9)	33.6	34.0
T2	34.3	33.5	36.3	27.9	37.9	30.0	35.5	31.0	35.4	16.7 (2.3)	34.6	17.0
T3	39.3	30.8	34.5	36.7	28.2	42.4	29.0	37.4	29.8	17.1 (2.8)	31.8	49.1
p-value	0.046*		0.126			0.004*		0.125		0.013*	0.012*	
**Fish**												
T1	32.0	33.7	28.6	37.6	31.3	34.8	33.9	28.2	37.2	16.4 (2.2)	34.8	20.8
T2	33.7	33.7	35.7	30.6	33.8	29.5	35.5	33.0	33.6	16.7 (2.2)	32.2	43.4
T3	34.3	32.6	35.7	31.9	34.9	35.7	30.6	38.8	29.2	17.1 (2.6)	33.0	35.8
p-value	0.897		0.415			0.274		0.017*		0.019*	0.091	
**Egg**												
T1	30.9	33.7	31.0	36.2	32.8	36.2	31.9	33.7	33.0	16.7 (2.4)	32.4	41.5
T2	37.1	31.9	36.3	31.4	31.3	34.3	33.4	33.0	33.3	16.6 (2.3)	33.7	32.1
T3	32.0	34.4	32.7	32.3	35.9	29.5	34.7	33.3	33.6	16.9 (2.4)	33.9	26.4
p-value	0.465		0.696			0.385		0.986		0.446	0.352	
**Nuts**												
T1	38.2	31.7	31.0	34.1	34.9	33.8	33.7	33.0	33.9	16.9 (2.6)	32.5	45.3
T2	29.2	35.1	36.3	32.8	29.7	33.3	32.4	31.6	34.5	16.6 (2.2)	33.0	30.2
T3	32.6	33.3	32.7	33.2	35.4	32.9	33.9	35.4	31.6	16.7 (2.2)	34.4	24.5

p-value	0.231		0.760			0.958		0.571		0.297	0.143	
**Butter**												
T1	50.0	26.5	33.3	34.9	29.7	45.2	27.5	33.0	33.3	17.0 (2.7)	32.5	47.2
T2	25.3	36.9	33.9	29.7	39.5	27.6	36.0	31.3	35.7	16.7 (2.4)	33.6	30.2
T3	24.7	36.6	32.7	35.4	30.8	27.1	36.5	35.7	31.0	16.5 (2)	33.9	22.6
p-value	<0.001*		0.338			<0.001*		0.372		0.098	0.077	
**Margarine**												
T1	21.3	37.8	28.0	35.4	32.3	27.6	35.5	35.0	31.3	16.5 (2.1)	34.8	18.9
T2	35.4	32.4	36.9	29.3	36.9	32.9	33.4	33.0	33.6	16.7 (2.2)	33.0	34.0
T3	43.3	29.9	35.1	35.4	30.8	39.5	31.1	32.0	35.1	17.1 (2.7)	32.2	47.2
p-value	<0.001*		0.303			0.066		0.562		0.044*	0.031*	
**Oil**												
T1	39.3	31.2	31.5	32.8	35.4	37.1	31.3	31.0	35.4	17.0 (2.5)	33.6	35.8
T2	36.5	31.9	29.8	34.9	33.3	32.9	34.2	36.1	31.6	16.8 (2.5)	33.7	34.0
T3	24.2	36.9	38.7	32.3	31.3	30.0	34.5	33.0	33.0	16.5 (2.0)	32.7	30.2
p-value	0.009*		0.569			0.323		0.391		0.093	0.919	
**Dairy**												
T1	36.0	32.8	38.1	34.5	29.2	39.5	29.8	32.3	35.1	16.7 (2.5)	32.2	47.2
T2	34.3	33.3	30.4	32.8	35.9	30.0	35.0	32.3	33.3	16.8 (2.3)	33.4	34.0
T3	29.8	33.9	31.5	32.8	34.9	30.5	35.2	35.4	31.6	16.7 (2.2)	34.4	18.9
p-value	0.582		0.505			0.055		0.577		0.786	0.033*	
**Sugar-sweetened food**												
T1	30.3	34.2	31.5	31.4	38.5	27.6	35.0	35.7	31.3	16.7 (2.2)	34.1	18.9
T2	32.6	33.7	28.0	37.6	30.8	31.9	34.2	33.3	33.3	16.9 (2.4)	33.0	39.6
T3	37.1	32.1	40.5	31.0	30.8	40.5	30.8	31.0	35.4	16.6 (2.4)	32.9	41.5
p-value	0.464		0.085			0.045*		0.393		0.447	0.077	
**Caloric drinks**												
T1	32.0	34.2	33.9	37.6	29.7	34.3	31.9	32.7	34.2	17.0 (2.5)	34.3	22.6
T2	27.5	35.3	35.7	31.0	32.8	31.4	35.0	35.7	31.3	16.7 (2.2)	34.1	28.3
T3	40.4	30.6	30.4	31.4	37.4	34.3	33.2	31.6	34.5	16.6 (2.3)	31.7	49.1
p-value	0.045*		0.376			0.670		0.486		0.282	0.032*	
**Tea [g/d]**												
T1	39.3	31.2	27.4	39.3	30.8	38.6	30.6	33.3	33.6	16.8 (2.4)	33.2	32.1
T2	32.0	33.7	33.3	33.2	33.8	31.9	35.5	34.4	32.4	16.8 (2.5)	33.2	37.7
T3	28.7	35.1	39.3	27.5	35.4	29.5	33.9	32.3	33.9	16.7 (2.2)	33.6	30.2
p-value	0.125		0.061			0.140		0.861		0.846	0.787	
**Water [g/d]**												
T1	39.3	31.7	31.5	30.1	39.0	34.3	32.4	33.7	32.7	16.2 (2.0)	31.8	50.9
T2	28.1	35.7	35.1	32.3	32.3	31.9	33.4	30.3	36.6	16.8 (2.6)	34.8	20.8
T3	32.6	32.6	33.3	37.6	28.7	33.8	34.2	36.1	30.7	17.2 (2.3)	33.4	28.3
p-value	0.111		0.243			0.883		0.195		<0.001*	0.014*	
**Protein**												
T1	33.1	33.9	35.7	31.9	31.8	32.9	34.7	32.0	34.8	16.1 (1.9)	33.0	37.7
T2	30.3	34.6	32.7	33.2	34.4	30.5	35.2	31.3	34.8	16.7 (2.3)	34.4	22.6
T3	36.5	31.5	31.5	34.9	33.8	36.7	30.1	36.7	30.4	17.4 (2.7)	32.5	39.6
p-value	0.426		0.915			0.235		0.238		<0.001*	0.215	
**Fat**												
T1	33.7	33.5	31.0	32.3	34.9	30.0	33.7	32.3	33.9	16.6 (2.3)	33.7	28.3
T2	33.7	32.6	31.5	31.9	39.0	31.9	35.5	32.0	34.8	16.9 (2.2)	33.6	32.1
T3	32.6	33.9	37.5	35.8	26.2	38.1	30.8	35.7	31.3	16.7 (2.5)	32.7	39.6
p-value	0.942		0.148			0.199		0.488		0.504	0.559	
**Carbohydrates**												
T1	34.8	33.0	36.9	38.0	25.1	36.2	32.4	35.7	31.9	16.9 (2.6)	33.2	35.8

T2	30.9	33.9	31.5	28.4	42.1	32.9	33.9	31.6	34.5	16.8 (2.2)	33.0	37.7
T3	34.3	33.0	31.5	33.6	32.8	31.0	33.7	32.7	33.6	16.5 (2.3)	33.7	26.4
p-value	0.765		0.015*			0.625		0.567		0.282	0.547	
**n3 PUFA**												
T1	31.5	33.9	31.0	33.6	34.4	30.5	33.9	28.6	36.9	16.5 (2.2)	32.5	37.7
T2	34.3	32.6	32.7	32.3	35.4	35.2	33.7	35.7	31.3	16.7 (2.2)	34.3	26.4
T3	34.3	33.5	36.3	34.1	30.3	34.3	32.4	35.7	31.9	17.0 (2.6)	33.2	35.8
p-value	0.833		0.789			0.689		0.086		0.076	0.499	
**n6 PUFA**												
T1	32.0	33.9	29.2	34.5	36.4	29.5	35.2	29.3	36.6	16.5 (2)	34.8	20.8
T2	31.5	33.7	30.4	29.3	37.4	33.8	33.7	33.0	33.6	16.9 (2.5)	33.0	37.7
T3	36.5	32.4	40.5	36.2	26.2	36.7	31.1	37.8	29.8	16.8 (2.5)	32.2	41.5
p-value	0.610		0.043*			0.270		0.062		0.206	0.109	
**Retinol [mg/d]**												
T1	25.8	36.2	29.8	28.8	40.0	27.6	35.2	33.7	33.9	16.7 (2.4)	33.2	34.0
T2	34.3	32.6	36.9	34.5	29.2	34.3	33.9	32	33.3	16.7 (2.3)	33.2	37.7
T3	39.9	31.2	33.3	36.7	30.8	38.1	30.8	34.4	32.7	16.8 (2.3)	33.6	28.3
p-value	0.030*		0.113			0.101		0.899		0.977	0.702	
**Beta Carotene [mg/d]**												
T1	42.1	29.9	37.5	31.0	34.4	38.1	31.1	28.9	38.1	16.9 (2.5)	31.5	54.7
T2	37.1	32.6	30.4	34.9	29.7	33.8	32.6	35.0	31.6	16.6 (2.2)	33.2	32.1
T3	20.8	37.5	32.1	34.1	35.9	28.1	36.3	36.1	30.4	16.7 (2.3)	35.3	13.2
p-value	<0.001*		0.616			0.092		0.05		0.382	0.001*	
**Vitamin C [mg/d]**												
T1	32.0	33.5	34.5	34.5	32.8	34.8	33.2	30.6	36.3	16.8 (2.2)	32.2	49.1
T2	34.8	32.6	33.3	35.8	30.8	34.8	31.3	36.7	30.1	16.7 (2.5)	33.9	22.6
T3	33.1	33.9	32.1	29.7	36.4	30.5	35.5	32.7	33.6	16.6 (2.3)	33.9	28.3
p-value	0.861		0.666			0.448		0.162		0.713	0.040*	
**alpha tocopherol [mg/d]**												
T1	34.3	32.1	34.5	30.6	33.8	32.9	33.2	29.6	36.6	16.7 (2.3)	33.4	35.8
T2	29.2	35.3	28.0	35.8	35.4	30.5	35.5	33.7	33.3	16.8 (2.4)	32.7	39.6
T3	36.5	32.6	37.5	33.6	30.8	36.7	31.3	36.7	30.1	16.7 (2.3)	33.9	24.5
p-value	0.340		0.422			0.337		0.110		0.944	0.354	

[1] Presented as percentage and tested using Pearson's χ^2 test for count data; [2] Presented as mean (standard deviation) and tested using one-way analysis of variance; *p-value <0·05

Supplementary Table 1b Associations between exposure variables and baseline food intake tertiles in males

	Parental educ.[1]		Family income level[1]			School level[1]		Puberty[1]		BMI[2]	Screen time[1]	
	lower	higher	lower	med	higher	lower	higher	yes	no		lower	higher
**N**	211	357	166	196	174	215	337	63	516	527	523	61
**Fruit**												
T1	39.3	30.8	41.0	33.2	27.6	38.6	29.4	36.5	33.1	16.8 (2.3)	31.0	52.5
T2	33.6	33.3	28.9	33.2	34.5	30.2	34.4	27.0	34.1	16.9 (2.3)	34.0	27.9
T3	27.0	35.9	30.1	33.7	37.9	31.2	36.2	36.5	32.8	16.7 (2.3)	35.0	19.7
p-value	0.049*		0.138			0.079		0.526		0.726	0.002*	
**Vegetable**												
T1	33.6	34.5	38.6	34.7	28.2	38.1	30.0	28.6	34.1	16.7 (2.3)	31.4	49.2
T2	38.4	30.3	33.7	31.6	32.2	33.5	33.5	36.5	32.8	16.9 (2.1)	33.8	27.9
T3	28.0	35.3	27.7	33.7	39.7	28.4	36.5	34.9	33.1	16.9 (2.5)	34.8	23.0
p-value	0.088		0.164			0.073		0.668		0.579	0.017*	
**Starchy vegetables**												
T1	27.0	37.5	28.9	34.2	35.6	30.2	35.9	49.2	31.4	16.4 (2.1)	34.0	29.5
T2	31.3	34.5	34.9	32.1	33.9	28.4	35.6	23.8	34.7	16.8 (2.1)	33.3	32.8
T3	41.7	28.0	36.1	33.7	30.5	41.4	28.5	27.0	33.9	17.4 (2.6)	32.7	37.7
p-value	0.002*		0.675			0.007*		0.017*		<0.001*	0.687	
**Wholegrain**												
T1	44.1	27.7	37.3	31.1	33.3	42.8	28.2	27.0	34.7	16.9 (2.3)	31.2	52.5
T2	33.2	33.3	31.3	33.2	33.9	31.6	34.1	31.7	32.9	16.8 (2.4)	34.6	24.6
T3	22.7	38.9	31.3	35.7	32.8	25.6	37.7	41.3	32.4	16.7 (2.1)	34.2	23.0
p-value	<0.001*		0.776			0.001*		0.310		0.742	0.004*	
**Refined grain**												
T1	33.2	33.6	30.1	33.2	32.8	34.4	32.6	28.6	34.1	16.7 (2.4)	33.8	31.1
T2	36.5	31.7	37.3	30.1	33.9	40.0	29.7	31.7	33.3	16.8 (2.2)	32.9	36.1
T3	30.3	34.7	32.5	36.7	33.3	25.6	37.7	39.7	32.6	17.0 (2.3)	33.3	32.8
p-value	0.425		0.691			0.006*		0.494		0.542	0.866	
**Meat**												
T1	27.5	36.4	33.7	35.2	31.0	27.0	37.1	30.2	33.3	16.3 (2.0)	34.0	29.5
T2	35.1	31.7	32.5	31.6	37.4	33.5	33.2	33.3	33.5	16.9 (2.4)	32.9	34.4
T3	37.4	31.9	33.7	33.2	31.6	39.5	29.7	36.5	33.1	17.3 (2.3)	33.1	36.1
p-value	0.089		0.805			0.020*		0.834		<0.001*	0.772	
**Fish**												
T1	32.7	33.6	33.1	31.1	33.9	35.3	32.0	34.9	32.8	16.6 (2.1)	33.3	31.1
T2	32.2	34.5	33.7	35.2	32.8	31.2	35.9	25.4	34.7	17.1 (2.4)	32.7	41.0
T3	35.1	31.9	33.1	33.7	33.3	33.5	32.0	39.7	32.6	16.8 (2.4)	34.0	27.9
p-value	0.732		0.982			0.502		0.305		0.086	0.404	
**Egg**												
T1	35.5	31.9	30.7	35.2	32.2	36.7	31.5	34.9	33.3	17.0 (2.4)	32.5	41.0
T2	30.8	35.3	31.9	34.2	32.8	33.0	33.2	30.2	33.3	16.6 (2.1)	33.8	27.9
T3	33.6	32.8	37.3	30.6	35.1	30.2	35.3	34.9	33.3	16.9 (2.4)	33.7	31.1
p-value	0.510		0.738			0.348		0.880		0.187	0.393	
**Nuts**												
T1	37.0	31.1	34.3	31.1	32.2	39.5	29.4	27.0	34.1	16.9 (2.4)	32.1	44.3
T2	31.3	33.9	30.1	35.2	33.3	32.1	34.1	38.1	33.1	16.9 (2.4)	33.8	27.9
T3	31.8	35.0	35.5	33.7	34.5	28.4	36.5	34.9	32.8	16.7 (2.1)	34.0	27.9
p-value	0.356		0.895			0.033*		0.511		0.822	0.164	
**Butter**												
T1	48.8	24.9	39.8	31.6	32.2	44.7	26.1	27.0	34.1	17.0 (2.4)	31.9	47.5
T2	29.4	35.6	31.9	32.7	32.2	31.6	33.8	44.4	32.0	16.9 (2.4)	33.3	31.1

T3	21.8	39.5	28.3	35.7	35.6	23.7	40.1	28.6	33.9	16.5 (2.0)	34.8	21.3
p-value	<0.001*		0.413			<0.001*		0.138		0.097	0.030*	
**Margarine**												
T1	23.2	38.7	27.7	39.3	35.1	23.3	40.4	38.1	32.8	16.7 (2.1)	34.4	23.0
T2	33.6	32.8	31.9	29.6	35.6	36.3	31.5	33.3	33.1	16.7 (2.2)	32.9	37.7
T3	43.1	28.6	40.4	31.1	29.3	40.5	28.2	28.6	34.1	17.1 (2.5)	32.7	39.3
p-value	<0.001*		0.085			<0.001*		0.608		0.189	0.195	
**Oil**												
T1	41.7	29.1	39.8	34.7	24.1	40.5	29.4	22.2	34.7	16.7 (2.3)	32.7	42.6
T2	33.6	33.1	33.1	30.6	38.5	34.0	32.6	38.1	32.9	17.0 (2.4)	32.7	37.7
T3	24.6	37.8	27.1	34.7	37.4	25.6	38.0	39.7	32.4	16.8 (2.2)	34.6	19.7
p-value	0.001*		0.024*			0.004*		0.137		0.539	0.058	
**Dairy**												
T1	37.0	31.9	34.9	33.2	31.6	34.0	32.6	41.3	32.9	16.9 (2.4)	32.3	42.6
T2	33.2	31.9	34.9	31.6	34.5	31.6	34.7	27.0	33.9	16.7 (2.3)	33.3	32.8
T3	29.9	36.1	30.1	35.2	33.9	34.4	32.6	31.7	33.1	16.9 (2.2)	34.4	24.6
p-value	0.272		0.848			0.753		0.369		0.617	0.190	
**Sugar-sweetened food**												
T1	26.1	37.3	25.9	35.2	41.4	30.2	36.2	41.3	32.6	17.1 (2.3)	34.2	27.9
T2	33.6	33.1	33.1	33.7	33.3	30.7	34.7	30.2	33.3	16.9 (2.1)	34.2	26.2
T3	40.3	29.7	41.0	31.1	25.3	39.1	29.1	28.6	34.1	16.5 (2.4)	31.5	45.9
p-value	0.009*		0.013*			0.050		0.376		0.057	0.078	
**Caloric drinks**												
T1	32.2	34.5	30.7	34.2	31.0	32.1	34.1	33.3	33.5	16.8 (2.3)	34.2	24.6
T2	32.7	33.1	37.3	31.6	32.8	31.6	34.4	38.1	32.8	16.9 (2.3)	32.5	39.3
T3	35.1	32.5	31.9	34.2	36.2	36.3	31.5	28.6	33.7	16.8 (2.3)	33.3	36.1
p-value	0.793		0.767			0.500		0.628		0.867	0.298	
**Tea [g/d]**												
T1	36.5	30.8	28.9	34.7	35.1	39.5	30.9	30.2	33.9	16.9 (2.4)	32.7	39.3
T2	34.6	33.6	37.3	28.6	32.8	33.5	32.3	30.2	33.7	16.9 (2.4)	33.1	36.1
T3	28.9	35.6	33.7	36.7	32.2	27.0	36.8	39.7	32.4	16.7 (2.1)	34.2	24.6
p-value	0.210		0.415			0.034*		0.507		0.595	0.302	
**Water [g/d]**												
T1	39.3	30.0	41.6	28.1	33.9	37.7	30.6	28.6	34.5	16.5 (2.3)	33.5	37.7
T2	30.3	35.0	28.9	38.3	33.3	28.8	36.8	34.9	32.9	16.8 (2.2)	33.1	34.4
T3	30.3	35.0	29.5	33.7	32.8	33.5	32.6	36.5	32.6	17.2 (2.4)	33.5	27.9
p-value	0.073		0.105			0.106		0.633		0.035*	0.657	
**Protein**												
T1	30.3	34.7	36.7	32.7	32.2	30.2	35.3	25.4	34.3	16.4 (2.1)	33.3	34.4
T2	34.1	33.3	33.7	33.7	33.3	33.0	33.8	30.2	33.9	16.7 (2.2)	32.5	41.0
T3	35.5	31.9	29.5	33.7	34.5	36.7	30.9	44.4	31.8	17.4 (2.5)	34.2	24.6
p-value	0.517		0.847			0.299		0.117		<0.001*	0.256	
**Fat**												
T1	34.1	32.2	29.5	38.8	31.6	29.8	34.7	28.6	33.7	16.6 (2.3)	33.3	34.4
T2	33.2	33.3	36.1	31.1	36.8	32.1	35.0	36.5	33.1	17.1 (2.4)	33.5	32.8
T3	32.7	34.5	34.3	30.1	31.6	38.1	30.3	34.9	33.1	16.8 (2.1)	33.3	32.8
p-value	0.874		0.387			0.153		0.707		0.083	0.984	
**Carbohydrates**												
T1	34.6	33.6	31.9	27.6	38.5	36.3	31.8	42.9	32.4	17.1 (2.3)	33.7	31.1
T2	33.2	33.6	34.9	38.3	28.7	35.8	32.0	33.3	33.5	16.9 (2.3)	33.3	34.4
T3	32.2	32.8	33.1	34.2	32.8	27.9	36.2	23.8	34.1	16.4 (2.2)	33.1	34.4
p-value	0.972		0.200			0.129		0.161		0.019*	0.926	
**n3 PUFA**												

T1	31.8	34.5	33.1	35.7	31.6	30.2	36.2	31.7	33.5	16.4 (1.9)	32.3	42.6
T2	30.3	35.0	35.5	31.6	33.3	31.6	32.3	34.9	33.1	16.9 (2.4)	34.0	27.9
T3	37.9	30.5	31.3	32.7	35.1	38.1	31.5	33.3	33.3	17.3 (2.4)	33.7	29.5
p-value	0.189		0.868			0.210		0.948		0.001*	0.267	
**n6 PUFA**												
T1	28.9	35.9	27.7	36.2	36.8	30.2	36.2	28.6	33.7	16.4 (2.1)	33.3	36.1
T2	36.5	31.7	35.5	32.1	28.7	33.0	32.0	31.7	33.7	16.8 (2.3)	32.9	36.1
T3	34.6	32.5	36.7	31.6	34.5	36.7	31.8	39.7	32.6	17.2 (2.4)	33.8	27.9
p-value	0.220		0.337			0.303		0.504		0.005*	0.644	
**Retinol [mg/d]**												
T1	32.7	34.2	33.1	32.7	35.1	34.0	33.2	31.7	33.1	16.5 (2.1)	32.9	37.7
T2	31.8	33.1	31.3	32.7	35.1	30.2	36.5	28.6	33.9	16.8 (2.4)	34.2	26.2
T3	35.5	32.8	35.5	34.7	29.9	35.8	30.3	39.7	32.9	17.1 (2.4)	32.9	36.1
p-value	0.796		0.824			0.249		0.530		0.045*	0.451	
**Beta Carotene [mg/d]**												
T1	39.3	30.3	34.3	30.6	34.5	43.7	26.7	27.0	33.7	16.7 (2.3)	31.5	47.5
T2	35.5	32.8	36.1	34.7	28.7	33.0	34.1	41.3	32.4	17.2 (2.2)	32.9	36.1
T3	25.1	37.0	29.5	34.7	36.8	23.3	39.2	31.7	33.9	16.6 (2.3)	35.6	16.4
p-value	0.010*		0.469			<0.001*		0.335		0.057	0.006*	
**Vitamin C [mg/d]**												
T1	37.0	31.7	36.1	37.2	28.2	37.7	31.2	30.2	33.9	16.6 (2.2)	32.7	36.1
T2	31.8	34.7	30.7	32.1	37.4	30.7	35.3	23.8	34.7	16.8 (2.1)	33.1	36.1
T3	31.3	33.6	33.1	30.6	34.5	31.6	33.5	46.0	31.4	17.1 (2.5)	34.2	27.9
p-value	0.431		0.369			0.268		0.053		0.073	0.609	
**alpha tocopherol [mg/d]**												
T1	36.5	31.4	31.3	38.8	31.6	35.8	33.2	31.7	33.5	16.6 (2.2)	32.7	37.7
T2	34.1	33.9	36.1	31.6	32.2	33.5	32.3	19.0	34.9	16.8 (2.3)	33.8	29.5
T3	29.4	34.7	32.5	29.6	36.2	30.7	34.4	49.2	31.6	17.1 (2.3)	33.5	32.8
p-value	0.332		0.432			0.651		0.009*		0.136	0.695	

[1] Presented as percentage and tested using Pearson's χ^2 test for count data; [2] Presented as mean (standard deviation) and tested using one-way analysis of variance; *p-value <0·05
