# Supplementary material for: Changes in dietary intake during puberty and their determinants: results from the GINIplus birth cohort study
Source: BMC Public Health. 2015 Sep 2;15:841. doi: 10.1186/s12889-015-2189-0 (PMC4556194; doi:10.1186/s12889-015-2189-0)
Supplement: Additional file 2: — Comparison of lost-to-follow-up and not-lost-to-follow-up participants (PDF 264 kb) [file 12889_2015_2189_MOESM2_ESM.pdf]

Additional file 2. Comparison of lost-to-follow-up and not-lost-to-follow-up participants

Table 2 Comparison of lost-to-follow-up and not-lost-to-follow-up participants

	LTF	NLTF	p-value
**N**	680	1232	
**Sex[1]**			
Boys	55.3	47.8	0.002*
Girls	44.7	52.2	
**Parental education level[1,3]**			
Low (≤ 10 years)	40.1	32.7	0.002*
High (> 10 years)	59.9	67.3	
**Family income level[1,4]**			
Lower	36.0	29.6	0.020*
Middle	32.8	37.7	
Higher	31.2	32.7	
**Child education level[1,3]**			
Low (≤ 10 years)	47.1	37.0	<0.001*
High (> 10 years)	52.9	63.0	
**Pubertal onset at BL[1]**			
Yes	27.0	29.5	0.287
No	73.0	70.5	
**Pubertal stage at FU[1]**			
Pre-pubertal	0.3	0.6	0.736
Early puberty	2.6	1.9	
Mid puberty	21.2	18.8	
Late puberty	67.2	70.6	
Post-pubertal	8.7	8.1	
**BMI [kg/m][2]**	17.0 (2.4)	16.8 (2.3)	0.071
**Screen-time at BL[1,5]**			
Low (≤ 2h)	85.1	90.6	0.002*
High (> 2h)	14.9	9.4	
**Age at BL [y][2]**	11.0 (0.5)	11.0 (0.5)	0.877
**Energy intake at BL [kcal/day][2]**	1975.1 (581.1)	1962.4 (542.2)	0.638
**Study center[1]**			
Munich	40.1	52.5	<0.001*
Wesel	59.9	47.5	
**Study arm[1]**			
Control group	54.9	55.0	1.000
Infant intervention	45.1	45.0	

LTF=lost-to-follow-up (data at baseline only); NLTF=not-lost-to-follow-up (data at baseline and follow-up); BL=baseline; FU=follow-up; [1]Presented as percentages, tested by Fisher's exact test (variables with 2 levels), or by Pearson's Chi^2 test (variables with > 2 levels) ; [2]Presented as mean (standard deviation), tested by t-test; [3]Highest level achieved by mother or father or achievable in the case of child education ;[4]Tertiles stratified by study centre and merged; [5] Hours spent on screen-behaviours; *p-value<0.05.
