# Supplementary material for: Changes in dietary intake during puberty and their determinants: results from the GINIplus birth cohort study
Source: BMC Public Health. 2015 Sep 2;15:841. doi: 10.1186/s12889-015-2189-0 (PMC4556194; doi:10.1186/s12889-015-2189-0)
Supplement: Additional file 3: — Associations with dietary intake changes stratified by baseline intake tertile (PDF 303 kb) [file 12889_2015_2189_MOESM3_ESM.pdf]

Additional file 3. Associations with dietary intake changes stratified by baseline intake tertile

Supplementary Table 3a Associations[1] with dietary intake changes stratified by baseline tertile in females

**Reference**	**Tracking in T1**[2]	**Tracking in T2**[3]		**Tracking in T3**[4]
**Change**	**Increase**	**Increase**	**Decrease**	**Decrease**
**Fruit**				
ParEdu high	0.6 (0.3;1.5)	1.1 (0.4;3.0)	1.0 (0.4;2.5)	0.5 (0.2;1.3)
Income med	2.0 (0.9;4.7)	1.0 (0.3;3.2)	1.1 (0.4;3.3)	1.2 (0.5;2.9)
Income high	1.9 (0.7;5.2)	0.8 (0.2;2.5)	1.0 (0.3;3.1)	1.0 (0.4;2.6)
ChildEdu high	1.9 (0.9;4.2)	0.9 (0.4;2.5)	0.8 (0.3;1.9)	1.7 (0.7;4.1)
Puberty yes	1.3 (0.7;2.7)	0.8 (0.3;2.0)	1.5 (0.7;3.4)	0.8 (0.4;1.5)
BMI	1.0 (0.8;1.1)	1.1 (0.9;1.3)	1.0 (0.8;1.2)	0.9 (0.8;1.0)
Sed high	1.2 (0.5;3.1)	0.8 (0.1;5.3)	1.6 (0.4;7.2)	2.6 (0.4;16.3)
**Vegetables**				
ParEdu high	1.3 (0.6;3.0)	0.5 (0.2;1.3)	0.3 (0.1;0.8)	0.8 (0.3;2.0)
Income med	0.4 (0.2;1.0)	2.2 (0.7;6.8)	1.2 (0.4;4.3)	0.6 (0.2;1.4)
Income high	0.4 (0.1;0.9)	1.7 (0.5;6.2)	2.0 (0.5;7.6)	0.5 (0.2;1.2)
ChildEdu high	0.8 (0.4;1.7)	1.1 (0.4;3.1)	1.3 (0.4;3.8)	0.5 (0.2;1.1)
Puberty yes	1.2 (0.6;2.4)	1.0 (0.4;2.2)	3.0 (1.3;7.4)	0.8 (0.4;1.5)
BMI	1.0 (0.9;1.2)	1.1 (0.9;1.3)	0.8 (0.7;1.1)	0.9 (0.8;1.1)
Sed high	0.5 (0.2;1.4)	1.5 (0.3;7.1)	1.4 (0.3;7.9)	0.4 (0.1;2.1)
**Starchy vegetables**				
ParEdu high	0.5 (0.2;1.4)	1.9 (0.6;5.7)	0.7 (0.3;2.0)	0.4 (0.1;0.9)
Income med	2.2 (0.9;5.7)	1.4 (0.4;4.4)	1.1 (0.4;3.1)	0.6 (0.2;1.6)
Income high	2.4 (0.9;7.0)	1.3 (0.4;4.2)	1.1 (0.4;3.0)	1.4 (0.5;4.1)
ChildEdu high	0.7 (0.3;1.6)	4.2 (1.4;12.0)	2.6 (1.0;6.5)	1.8 (0.8;4.1)
Puberty yes	0.7 (0.4;1.4)	0.8 (0.3;1.9)	0.4 (0.2;0.9)	1.0 (0.4;2.1)
BMI	1.0 (0.9;1.2)	1.2 (0.9;1.5)	1.0 (0.8;1.3)	1.0 (0.8;1.1)
Sed high	1.5 (0.4;5.3)	0.8 (0.2;4.2)	0.9 (0.2;4.2)	0.9 (0.3;3.0)
**Refined grain**				
ParEdu high	1.7 (0.7;4.0)	1.5 (0.5;4.2)	1.3 (0.5;3.5)	0.9 (0.4;2.1)
Income med	0.8 (0.3;2.1)	1.3 (0.4;4.3)	0.5 (0.2;1.4)	1.4 (0.6;3.4)
Income high	0.5 (0.2;1.4)	0.6 (0.2;2.2)	0.4 (0.1;1.4)	1.0 (0.4;2.6)
ChildEdu high	2.3 (1.0;5.1)	0.9 (0.3;2.3)	0.5 (0.2;1.2)	0.9 (0.4;1.9)
Puberty yes	1.0 (0.5;2.1)	1.2 (0.5;2.9)	1.2 (0.5;2.7)	0.7 (0.4;1.5)
BMI	1.0 (0.8;1.1)	1.1 (0.9;1.3)	1.0 (0.9;1.2)	1.0 (0.8;1.1)
Sed high	2.6 (0.7;9.6)	2.1 (0.4;11.1)	2.1 (0.5;9.3)	1.9 (0.6;6.1)
**Meat**				
ParEdu high	1.0 (0.4;2.5)	1.7 (0.6;4.2)	1.3 (0.5;3.3)	0.9 (0.4;1.9)
Income med	1.1 (0.4;2.7)	0.8 (0.3;2.2)	1.6 (0.5;4.8)	0.4 (0.2;1.0)
Income high	1.4 (0.5;3.7)	0.7 (0.2;2.0)	1.5 (0.5;4.6)	0.4 (0.1;1.1)
ChildEdu high	0.4 (0.2;0.9)	0.5 (0.2;1.2)	0.5 (0.2;1.4)	1.4 (0.6;3.1)
Puberty yes	1.7 (0.8;3.5)	1.1 (0.5;2.4)	1.4 (0.6;3.2)	1.4 (0.7;3.0)
BMI	1.0 (0.8;1.2)	1.1 (0.9;1.3)	1.0 (0.8;1.2)	0.9 (0.8;1.0)
Sed high	2.3 (0.6;8.3)	0.2 (0.0;1.7)	0.6 (0.1;3.0)	0.4 (0.1;1.3)
**Fish**				
ParEdu high	1.2 (0.5;2.8)	0.7 (0.3;1.9)	0.8 (0.3;2.2)	0.4 (0.2;1.0)
Income med	0.6 (0.3;1.5)	2.1 (0.7;6.2)	1.2 (0.4;3.4)	1.9 (0.7;5.0)
Income high	0.5 (0.2;1.3)	1.8 (0.6;5.2)	1.2 (0.4;3.4)	1.8 (0.6;4.9)
ChildEdu high	1.2 (0.6;2.7)	0.7 (0.3;1.8)	0.8 (0.3;2.0)	1.2 (0.5;2.7)
Puberty yes	0.8 (0.4;1.5)	0.8 (0.3;1.8)	0.9 (0.4;1.9)	0.6 (0.3;1.2)
BMI	1.1 (0.9;1.3)	1.1 (0.9;1.3)	1.2 (0.9;1.4)	1.1 (0.9;1.2)
Sed high	1.6 (0.4;6.5)	1.6 (0.4;5.9)	0.5 (0.1;2.2)	2.2 (0.7;7.2)

**Egg**				
ParEdu high	0.8 (0.4;1.9)	1.5 (0.5;4.2)	1.7 (0.6;4.5)	0.5 (0.2;1.3)
Income med	0.6 (0.3;1.6)	2.2 (0.8;6.0)	2.0 (0.7;5.5)	1.2 (0.5;2.9)
Income high	0.8 (0.3;2.0)	3.2 (1.0;10.4)	2.6 (0.8;8.4)	0.6 (0.2;1.6)
ChildEdu high	1.1 (0.5;2.3)	0.3 (0.1;0.8)	0.5 (0.2;1.3)	1.7 (0.7;3.9)
Puberty yes	1.6 (0.8;3.2)	1.1 (0.5;2.7)	0.6 (0.3;1.5)	1.7 (0.8;3.4)
BMI	1.1 (0.9;1.3)	1.2 (1.0;1.5)	1.1 (0.9;1.4)	1.0 (0.9;1.2)
Sed high	0.8 (0.3;2.3)	2.2 (0.5;10.3)	1.8 (0.4;8.7)	0.4 (0.1;1.8)
**Butter**				
ParEdu high	0.9 (0.4;2.0)	0.7 (0.2;2.0)	0.8 (0.3;2.6)	0.9 (0.3;2.2)
Income med	3.5 (1.3;9.2)	0.5 (0.1;1.4)	1.3 (0.4;3.9)	0.7 (0.3;1.7)
Income high	1.3 (0.4;4.1)	0.9 (0.3;2.8)	1.6 (0.5;5.5)	1.3 (0.5;3.3)
ChildEdu high	0.6 (0.3;1.3)	2.4 (0.8;7.4)	1.0 (0.4;2.5)	0.6 (0.3;1.4)
Puberty yes	1.9 (0.9;4.1)	1.0 (0.4;2.4)	0.8 (0.3;1.8)	1.0 (0.5;2.1)
BMI	1.0 (0.8;1.1)	0.9 (0.7;1.1)	1.0 (0.9;1.3)	1.0 (0.9;1.3)
Sed high	0.8 (0.2;2.7)	1.0 (0.2;4.9)	0.9 (0.2;3.9)	2.0 (0.5;7.4)
**Margarine**				
ParEdu high	0.3 (0.1;0.7)	0.9 (0.3;2.2)	1.7 (0.5;5.6)	1.0 (0.5;2.1)
Income med	1.4 (0.5;3.8)	0.8 (0.3;2.3)	0.3 (0.1;1.1)	0.7 (0.3;1.6)
Income high	1.1 (0.3;3.4)	0.2 (0.1;0.8)	0.3 (0.1;0.8)	1.1 (0.4;2.9)
ChildEdu high	0.8 (0.3;2.0)	1.6 (0.6;4.3)	1.2 (0.4;3.5)	0.8 (0.3;1.6)
Puberty yes	0.9 (0.4;1.9)	1.1 (0.5;2.4)	1.5 (0.6;3.7)	1.9 (1.0;3.9)
BMI	1.0 (0.8;1.2)	1.1 (0.9;1.3)	1.0 (0.8;1.3)	0.9 (0.8;1.0)
Sed high	0.6 (0.1;3.5)	1.9 (0.5;7.2)	0.3 (0.0;2.5)	0.9 (0.3;2.7)
**Oil**				
ParEdu high	1.3 (0.6;2.9)	0.9 (0.3;2.3)	0.8 (0.3;2.0)	1.3 (0.5;3.2)
Income med	1.8 (0.7;4.5)	0.9 (0.3;2.8)	2.1 (0.6;7.0)	0.9 (0.4;2.0)
Income high	1.3 (0.5;3.6)	0.8 (0.2;2.8)	2.0 (0.5;7.6)	0.8 (0.3;1.9)
ChildEdu high	1.4 (0.6;2.8)	1.3 (0.5;3.5)	0.8 (0.3;2.2)	0.8 (0.3;1.8)
Puberty yes	1.7 (0.8;3.5)	0.9 (0.4;2.2)	0.9 (0.4;2.1)	1.3 (0.6;2.5)
BMI	1.0 (0.9;1.2)	1.1 (0.9;1.3)	1.0 (0.8;1.2)	1.1 (0.9;1.3)
Sed high	0.6 (0.2;2.0)	3.6 (0.8;16.6)	0.8 (0.1;5.3)	1.0 (0.3;3.0)
**Dairy**				
ParEdu high	1.1 (0.5;2.4)	1.0 (0.4;2.7)	0.9 (0.3;2.5)	0.4 (0.2;1.0)
Income med	0.9 (0.4;2.0)	0.7 (0.2;2.0)	0.8 (0.2;2.4)	0.6 (0.2;1.4)
Income high	0.4 (0.1;0.9)	0.3 (0.1;0.9)	0.5 (0.1;1.7)	0.5 (0.2;1.4)
ChildEdu high	1.4 (0.6;2.9)	1.8 (0.6;5.2)	1.1 (0.4;3.3)	0.9 (0.4;2.0)
Puberty yes	1.4 (0.7;2.7)	0.9 (0.4;2.0)	0.5 (0.2;1.3)	1.1 (0.5;2.3)
BMI	1.0 (0.9;1.1)	1.1 (0.9;1.3)	1.1 (0.9;1.3)	0.8 (0.7;1.0)
Sed high	0.9 (0.3;2.4)	0.8 (0.1;4.3)	2.9 (0.7;12.0)	1.8 (0.3;10.4)
**Sugar-sweetened food**				
ParEdu high	0.8 (0.4;2.0)	0.3 (0.1;0.9)	1.2 (0.4;3.7)	0.8 (0.3;1.7)
Income med	1.3 (0.5;3.3)	2.9 (0.9;9.1)	1.1 (0.3;3.5)	3.1 (1.3;7.5)
Income high	1.0 (0.4;2.7)	2.5 (0.7;9.2)	1.7 (0.5;6.0)	1.3 (0.5;3.2)
ChildEdu high	0.9 (0.4;2.1)	0.6 (0.2;1.6)	0.4 (0.1;1.1)	0.6 (0.3;1.3)
Puberty yes	1.7 (0.8;3.3)	0.6 (0.2;1.4)	0.7 (0.3;1.8)	1.8 (0.9;3.5)
BMI	1.0 (0.8;1.2)	1.0 (0.8;1.2)	1.0 (0.8;1.2)	1.1 (1.0;1.3)
Sed high	0.7 (0.1;3.7)	1.3 (0.3;5.5)	1.7 (0.4;6.7)	0.6 (0.2;1.9)
**Caloric drinks**				
ParEdu high	0.7 (0.3;1.7)	1.1 (0.4;2.8)	2.3 (0.7;7.5)	0.4 (0.2;0.9)
Income med	1.1 (0.4;2.7)	0.9 (0.3;2.6)	1.1 (0.4;3.4)	2.3 (0.9;5.9)
Income high	1.9 (0.7;5.6)	0.9 (0.3;2.6)	1.4 (0.4;4.3)	0.9 (0.3;2.4)
ChildEdu high	0.6 (0.2;1.3)	0.3 (0.1;0.8)	0.6 (0.2;1.8)	2.3 (1.0;5.4)
Puberty yes	1.3 (0.7;2.7)	0.8 (0.4;1.9)	1.3 (0.5;2.9)	0.9 (0.4;1.8)
BMI	1.0 (0.9;1.2)	1.0 (0.9;1.3)	1.0 (0.8;1.2)	1.1 (0.9;1.2)
Sed high	0.6 (0.1;2.7)	1.3 (0.2;7.8)	2.0 (0.3;12.3)	1.1 (0.4;3.3)

**Tea [ml/d]**				
ParEdu high	1.8 (0.8;4.3)	1.0 (0.3;2.8)	0.6 (0.2;1.6)	0.8 (0.3;2.1)
Income med	0.6 (0.2;1.7)	1.3 (0.4;3.8)	0.6 (0.2;1.9)	0.7 (0.3;1.7)
Income high	0.7 (0.2;2.2)	0.9 (0.3;2.9)	0.5 (0.1;1.7)	0.7 (0.3;1.8)
ChildEdu high	1.5 (0.7;3.4)	0.9 (0.3;2.5)	1.0 (0.4;2.7)	0.8 (0.3;1.8)
Puberty yes	1.7 (0.8;3.5)	2.2 (1.0;5.0)	1.0 (0.4;2.4)	1.1 (0.5;2.2)
BMI	1.0 (0.8;1.1)	0.8 (0.7;1.0)	1.0 (0.9;1.2)	1.0 (0.9;1.2)
Sed high	0.9 (0.3;3.3)	1.0 (0.3;3.6)	0.3 (0.1;1.8)	3.5 (0.8;15.4)
**Water [ml/d]**				
ParEdu high	0.8 (0.3;1.7)	1.0 (0.3;2.8)	1.0 (0.4;3.1)	1.6 (0.6;3.9)
Income med	1.3 (0.5;3.1)	1.0 (0.3;2.8)	0.5 (0.2;1.4)	0.8 (0.3;2.0)
Income high	0.9 (0.3;2.2)	0.9 (0.3;3.0)	1.3 (0.5;4.0)	0.8 (0.3;2.4)
ChildEdu high	1.7 (0.8;3.9)	1.1 (0.4;2.9)	0.5 (0.2;1.3)	0.4 (0.2;1.0)
Puberty yes	1.2 (0.6;2.4)	1.3 (0.5;3.1)	1.7 (0.7;4.1)	0.9 (0.4;1.8)
BMI	1.1 (0.9;1.3)	1.1 (0.9;1.3)	1.1 (0.9;1.3)	0.9 (0.8;1.1)
Sed high	0.7 (0.3;2.0)	3.6 (0.3;41.4)	2.7 (0.3;29.8)	0.6 (0.2;2.5)
**Fat**				
ParEdu high	0.7 (0.3;1.4)	0.5 (0.2;1.5)	0.6 (0.2;1.8)	0.7 (0.3;1.7)
Income med	1.1 (0.5;2.6)	2.2 (0.7;6.5)	1.9 (0.6;6.3)	0.8 (0.4;1.8)
Income high	0.8 (0.3;2.1)	2.6 (0.8;8.4)	2.0 (0.5;7.2)	1.1 (0.4;3.0)
ChildEdu high	0.9 (0.4;2.0)	1.2 (0.5;2.9)	1.1 (0.4;3.1)	0.8 (0.4;1.6)
Puberty yes	0.9 (0.5;1.8)	0.8 (0.3;1.7)	0.4 (0.2;1.0)	1.0 (0.5;2.0)
BMI	0.9 (0.8;1.1)	1.1 (0.9;1.3)	1.1 (0.9;1.4)	1.0 (0.9;1.2)
Sed high	0.7 (0.2;2.4)	2.3 (0.5;10.7)	2.6 (0.5;13.2)	1.6 (0.5;5.4)
**Carbohydrate**				
ParEdu high	0.8 (0.3;1.9)	0.4 (0.1;1.2)	0.6 (0.2;1.6)	1.2 (0.5;2.6)
Income med	0.7 (0.3;1.7)	2.6 (0.8;8.3)	1.1 (0.3;3.4)	1.5 (0.6;3.5)
Income high	0.8 (0.3;2.0)	2.1 (0.6;7.2)	1.3 (0.4;4.0)	0.7 (0.3;1.9)
ChildEdu high	1.6 (0.7;3.5)	1.0 (0.4;2.5)	2.5 (0.9;6.4)	0.6 (0.3;1.4)
Puberty yes	1.6 (0.8;3.2)	1.1 (0.5;2.6)	1.5 (0.7;3.4)	1.1 (0.5;2.2)
BMI	1.0 (0.8;1.1)	1.0 (0.8;1.3)	1.2 (1.0;1.4)	1.0 (0.9;1.2)
Sed high	1.1 (0.3;3.8)	2.6 (0.5;12.7)	2.2 (0.5;9.8)	0.4 (0.1;1.6)
**n3PUFA**				
ParEdu high	0.8 (0.4;1.8)	0.9 (0.3;2.5)	0.5 (0.2;1.3)	0.4 (0.2;0.9)
Income med	0.8 (0.3;2.0)	1.0 (0.4;3.0)	2.4 (0.8;7.1)	0.6 (0.3;1.4)
Income high	1.0 (0.4;2.7)	1.1 (0.4;3.2)	1.3 (0.4;4.1)	0.7 (0.3;1.7)
ChildEdu high	1.3 (0.6;2.7)	1.0 (0.4;2.8)	0.5 (0.2;1.2)	1.8 (0.8;4.0)
Puberty yes	1.1 (0.6;2.3)	1.9 (0.8;4.4)	0.7 (0.3;1.6)	0.5 (0.2;1.0)
BMI	1.0 (0.8;1.1)	0.8 (0.7;1.0)	0.9 (0.8;1.1)	1.0 (0.9;1.2)
Sed high	1.0 (0.3;2.8)	0.3 (0.0;2.3)	0.3 (0.1;2.1)	0.7 (0.2;2.2)
**n6PUFA**				
ParEdu high	1.0 (0.4;2.3)	2.0 (0.7;5.7)	0.6 (0.2;1.7)	1.1 (0.5;2.6)
Income med	1.5 (0.6;3.6)	1.1 (0.4;3.3)	2.2 (0.7;7.2)	0.6 (0.3;1.4)
Income high	1.0 (0.4;2.6)	0.4 (0.1;1.3)	1.4 (0.4;4.5)	0.8 (0.3;2.2)
ChildEdu high	0.6 (0.2;1.3)	0.6 (0.2;1.5)	0.7 (0.3;2.1)	2.4 (1.0;5.3)
Puberty yes	1.0 (0.5;2.0)	0.9 (0.4;2.0)	0.8 (0.3;1.8)	1.1 (0.5;2.2)
BMI	1.1 (0.9;1.4)	0.9 (0.8;1.1)	1.0 (0.8;1.1)	1.1 (1.0;1.3)
Sed high	0.3 (0.1;1.5)	1.3 (0.4;4.6)	1.1 (0.2;5.1)	0.5 (0.2;1.7)
**Beta-Carotene [ml/d]**				
ParEdu high	1.8 (0.8;4.0)	1.3 (0.5;3.3)	0.8 (0.3;2.0)	1.5 (0.6;4.0)
Income med	1.1 (0.5;2.5)	0.4 (0.2;1.3)	1.4 (0.4;4.8)	0.4 (0.2;1.0)
Income high	1.0 (0.4;2.4)	0.3 (0.1;0.9)	1.8 (0.5;6.5)	0.4 (0.1;1.0)
ChildEdu high	0.8 (0.4;1.7)	0.8 (0.3;2.1)	0.6 (0.2;1.6)	0.7 (0.3;1.5)
Puberty yes	1.1 (0.5;2.1)	1.2 (0.5;2.7)	1.4 (0.6;3.2)	1.1 (0.6;2.2)
BMI	0.9 (0.8;1.1)	1.0 (0.8;1.2)	0.9 (0.8;1.2)	1.0 (0.9;1.2)
Sed high	0.9 (0.3;2.2)	1.2 (0.2;6.4)	3.3 (0.7;14.2)	4.5 (0.4;45.6)

**Alpha-Tocopherol [ml/d]**				
ParEdu high	1.9 (0.8;4.3)	1.9 (0.6;5.7)	0.3 (0.1;0.9)	1.3 (0.6;2.8)
Income med	0.9 (0.4;2.3)	1.9 (0.6;5.9)	1.0 (0.4;2.9)	0.6 (0.3;1.4)
Income high	0.6 (0.2;1.5)	1.4 (0.4;4.6)	0.5 (0.1;1.6)	0.6 (0.3;1.5)
ChildEdu high	0.7 (0.3;1.6)	1.1 (0.4;2.8)	1.1 (0.4;2.9)	1.3 (0.6;2.7)
Puberty yes	0.8 (0.4;1.6)	1.5 (0.7;3.4)	1.4 (0.6;3.3)	0.9 (0.4;1.6)
BMI	0.9 (0.8;1.1)	0.9 (0.7;1.0)	0.9 (0.8;1.1)	1.0 (0.9;1.1)
Sed high	0.4 (0.1;1.4)	0.6 (0.1;2.2)	1.0 (0.3;3.4)	1.3 (0.3;4.6)

[1]Odds ratio (95% CI); [2] Logistic regression (increase vs. tracking in lowest tertile). [3]Multinomial logistic regression (increase or decrease vs. tracking in medium tertile), [4]Logsitic regression (decrease vs. tracking in highest tertile); ParEdu high: parental education (high vs. low); Income med/high: family income (medium/high vs. low); ChildEdu high: child education (high vs. low); Puberty yes: pubertal onset at baseline (yes vs. no); Screen high: screen-time at baseline (high vs. low).*p-value < 0.0083 (Bonferroni correction for multiple testing: 0.05/6)

Supplementary Table 3b Associations[1] with dietary intake changes stratified by baseline tertile in males

Reference	Tracking in T1[2]	Tracking in T2[3]		Tracking in T3[4]
Change	Increase	Increase	Decrease	Decrease
**Fruit**				
ParEdu High	0.8 (0.4;1.9)	1.3 (0.4;4.3)	1.1 (0.3;3.8)	0.8 (0.3;2.1)
Income med	0.9 (0.4;2.2)	0.6 (0.2;1.8)	0.7 (0.2;2.3)	1.2 (0.5;3.2)
Income high	0.6 (0.2;1.5)	0.7 (0.2;2.7)	0.8 (0.2;3.1)	1.1 (0.4;2.9)
School high	2.0 (0.9;4.7)	0.3 (0.1;0.9)	0.8 (0.2;2.5)	0.8 (0.3;2.0)
Puberty yes	0.4 (0.1;1.4)	0.5 (0.1;2.8)	1.6 (0.3;8.9)	1.3 (0.4;3.9)
BMI	1.0 (0.9;1.2)	1.0 (0.8;1.3)	0.9 (0.7;1.1)	0.8 (0.7;1.0)
Sed high	0.6 (0.2;1.7)	3.7 (0.9;16.1)	0.7 (0.1;4.6)	2.3 (0.6;8.9)
**Vegetables[5]**				
ParEdu High	1.0 (0.4;2.6)	1.3 (0.5;4.0)	2.5 (0.8;8.2)	2.0 (0.8;5.4)
Income med	2.0 (0.8;5.4)	0.8 (0.3;2.4)	0.8 (0.3;2.8)	1.4 (0.5;3.5)
Income high	3.2 (1.1;9.4)	0.4 (0.1;1.5)	0.6 (0.2;2.2)	0.7 (0.3;1.9)
School high	1.0 (0.4;2.5)	1.9 (0.6;5.4)	2.0 (0.6;6.5)	0.9 (0.3;2.1)
Puberty yes	0.4 (0.1;1.4)	0.5 (0.1;1.9)	1.7 (0.4;7.1)	1.3 (0.4;4.2)
BMI	1.0 (0.9;1.2)	1.2 (1.0;1.5)	0.9 (0.7;1.1)	0.9 (0.8;1.1)
Sed high	0.3 (0.1;0.9)	1.9 (0.3;11.7)	8.3 (1.4;50.0)	2.5 (0.6;10.7)
**Starchy vegetables[5,6]**				
ParEdu High	1.3 (0.5;3.5)	0.3 (0.1;1.0)	0.4 (0.1;1.6)	0.7 (0.3;1.6)
Income med	0.6 (0.3;1.7)	1.4 (0.5;4.1)	2.9 (0.8;10.2)	1.7 (0.7;4.1)
Income high	0.4 (0.2;1.2)	2.1 (0.6;7.0)	3.3 (0.8;13.1)	2.4 (0.9;6.7)
School high	1.0 (0.4;2.5)	0.8 (0.3;2.4)	0.8 (0.2;3.0)	2.0 (0.9;4.6)
Puberty yes	0.5 (0.2;1.7)	-	-	2.9 (0.8;10.8)
BMI	1.0 (0.8;1.2)	0.8 (0.7;1.0)	0.8 (0.6;1.0)	1.0 (0.8;1.1)
Sed high	0.5 (0.2;1.9)	0.4 (0.1;1.9)	0.7 (0.1;3.1)	1.3 (0.5;4.0)
**Wholegrain**				
ParEdu High	1.3 (0.6;3.0)	2.5 (0.8;8.0)	1.4 (0.5;4.4)	1.7 (0.6;4.6)
Income med	1.3 (0.5;3.1)	0.6 (0.2;1.8)	0.6 (0.2;2.0)	0.6 (0.2;1.5)
Income high	1.3 (0.5;3.3)	1.2 (0.3;4.1)	0.7 (0.2;2.4)	0.9 (0.3;2.7)
School high	1.4 (0.6;3.4)	1.1 (0.4;3.4)	0.7 (0.2;1.9)	0.3 (0.1;0.9)
Puberty yes	0.9 (0.3;3.0)	5.6 (0.5;60.3)	18.8 (1.9;183.0)	1.1 (0.4;3.3)
BMI	1.1 (1.0;1.3)	1.1 (0.9;1.3)	0.8 (0.6;1.0)	1.0 (0.8;1.2)
Sed high	1.0 (0.4;2.5)	1.3 (0.2;7.3)	0.9 (0.2;4.7)	10.5 (1.6;67.3)
**Refined grain[5]**				
ParEdu High	1.2 (0.4;3.4)	1.0 (0.4;2.7)	1.2 (0.5;3.2)	0.8 (0.3;1.9)
Income med	2.2 (0.8;6.1)	1.3 (0.5;3.8)	0.7 (0.3;2.0)	0.9 (0.4;2.2)
Income high	4.4 (1.3;14.8)	0.9 (0.3;2.8)	0.3 (0.1;1.0)	1.6 (0.6;4.1)
School high	1.3 (0.5;3.6)	0.9 (0.3;2.2)	0.9 (0.3;2.2)	1.4 (0.6;3.4)
Puberty yes	2.9 (0.7;12.7)	1.0 (0.2;4.3)	0.6 (0.1;2.6)	1.6 (0.6;4.5)
BMI	0.9 (0.7;1.0)	1.0 (0.9;1.2)	1.0 (0.9;1.2)	1.1 (0.9;1.2)
Sed high	1.4 (0.4;4.3)	1.0 (0.2;4.2)	1.5 (0.4;5.4)	1.1 (0.3;3.6)
**Meat[5]**				
ParEdu High	0.2 (0.1;0.7)	1.9 (0.7;5.3)	2.5 (0.9;7.5)	1.3 (0.5;3.1)
Income med	2.4 (0.9;6.4)	1.3 (0.4;4.2)	1.1 (0.3;3.9)	1.9 (0.8;4.7)
Income high	0.7 (0.2;2.3)	0.5 (0.2;1.7)	0.6 (0.2;2.0)	1.2 (0.4;3.2)
School high	2.9 (1.0;8.2)	0.7 (0.3;2.0)	0.8 (0.3;2.3)	1.3 (0.5;3.1)
Puberty yes	1.2 (0.3;4.5)	0.6 (0.1;2.3)	0.6 (0.1;2.5)	0.7 (0.2;2.0)
BMI	1.1 (0.9;1.4)	1.1 (0.9;1.3)	1.0 (0.8;1.2)	1.1 (1.0;1.4)
Sed high	2.2 (0.6;8.6)	2.7 (0.6;12.1)	4.3 (0.9;19.2)	0.2 (0.1;0.7)
**Fish**				
ParEdu High	0.8 (0.3;2.0)	1.6 (0.6;4.6)	2.3 (0.8;7.0)	0.7 (0.3;1.8)
Income med	0.6 (0.2;1.6)	0.4 (0.1;1.3)	0.5 (0.2;1.5)	0.8 (0.3;2.0)
Income high	0.6 (0.2;1.7)	0.6 (0.2;1.8)	0.2 (0.1;0.8)	1.2 (0.4;3.2)
School high	1.4 (0.5;3.8)	0.8 (0.3;2.1)	0.8 (0.3;2.4)	1.7 (0.7;4.3)

Puberty yes	1.2 (0.3;4.3)	1.8 (0.5;7.2)	0.7 (0.1;3.5)	0.9 (0.3;2.6)
BMI	1.1 (0.9;1.4)	1.0 (0.8;1.1)	1.0 (0.8;1.2)	1.3 (1.0;1.6)
Sed high	1.1 (0.3;3.6)	0.9 (0.3;3.1)	0.9 (0.3;3.0)	0.9 (0.2;3.7)
**Nuts**[5]				
ParEdu High	1.8 (0.7;4.4)	0.7 (0.2;2.1)	1.3 (0.4;4.0)	1.1 (0.4;2.8)
Income med	0.5 (0.2;1.3)	1.0 (0.3;2.7)	1.3 (0.4;4.1)	0.6 (0.2;1.5)
Income high	0.6 (0.2;1.7)	0.9 (0.3;2.7)	1.2 (0.4;4.1)	1.1 (0.4;2.8)
School high	0.6 (0.2;1.4)	2.5 (0.9;6.9)	1.2 (0.4;3.5)	1.3 (0.5;3.3)
Puberty yes	0.3 (0.1;1.5)	1.3 (0.4;4.5)	1.6 (0.5;5.7)	1.5 (0.5;4.5)
BMI	1.1 (0.9;1.3)	1.1 (0.9;1.3)	1.1 (0.9;1.3)	0.8 (0.7;1.0)
Sed high	0.5 (0.2;1.5)	1.0 (0.3;4.3)	1.8 (0.4;7.2)	1.0 (0.3;3.8)
**Butter**[5]				
ParEdu High	1.1 (0.5;2.9)	1.3 (0.4;3.9)	0.5 (0.2;1.6)	0.9 (0.3;2.7)
Income med	1.2 (0.4;3.1)	2.2 (0.7;7.3)	0.5 (0.1;1.6)	1.4 (0.5;4.0)
Income high	1.1 (0.4;3.3)	2.0 (0.6;7.0)	0.6 (0.2;2.4)	0.5 (0.2;1.4)
School high	1.9 (0.8;4.7)	1.4 (0.4;4.3)	4.2 (1.2;15.4)	0.5 (0.2;1.5)
Puberty yes	1.7 (0.5;5.9)	0.6 (0.2;2.3)	0.6 (0.1;3.1)	0.8 (0.2;2.8)
BMI	1.2 (1.0;1.4)	0.9 (0.8;1.1)	0.9 (0.8;1.2)	1.0 (0.8;1.2)
Sed high	0.8 (0.3;2.3)	1.0 (0.2;4.4)	1.2 (0.3;5.4)	0.3 (0.1;1.8)
**Oil**				
ParEdu High	2.0 (0.8;5.0)	0.6 (0.2;1.9)	0.4 (0.1;1.4)	2.1 (0.8;5.6)
Income med	2.7 (1.1;6.6)	1.7 (0.5;5.5)	1.0 (0.3;3.1)	3.7 (1.3;10.2)
Income high	2.2 (0.8;6.3)	1.1 (0.3;3.3)	0.4 (0.1;1.5)	1.6 (0.6;4.5)
School high	0.7 (0.3;1.6)	1.5 (0.5;4.7)	3.1 (0.9;10.0)	0.5 (0.2;1.3)
Puberty yes	1.5 (0.4;6.1)	3.0 (0.6;14.7)	6.8 (1.2;37.2)	1.0 (0.3;3.3)
BMI	1.0 (0.9;1.2)	1.0 (0.8;1.2)	1.1 (0.9;1.3)	1.2 (1.0;1.5)
Sed high	2.2 (0.7;6.7)	0.2 (0.1;0.9)	0.8 (0.2;2.5)	1.1 (0.2;6.1)
**Dairy**				
ParEdu High	1.4 (0.6;3.5)	1.7 (0.5;5.7)	1.0 (0.3;3.2)	0.6 (0.2;1.5)
Income med	1.3 (0.5;3.2)	0.9 (0.3;2.8)	0.5 (0.1;1.5)	1.7 (0.6;4.8)
Income high	1.1 (0.4;3.0)	0.4 (0.1;1.4)	0.3 (0.1;1.0)	1.5 (0.5;4.4)
School high	0.5 (0.2;1.2)	1.2 (0.4;3.4)	2.4 (0.8;7.0)	1.2 (0.5;3.0)
Puberty yes	2.8 (0.9;8.5)	4.3 (0.7;27.7)	4.7 (0.7;31.4)	0.7 (0.2;2.2)
BMI	0.9 (0.8;1.1)	0.9 (0.8;1.1)	0.9 (0.8;1.1)	1.0 (0.9;1.2)
Sed high	0.4 (0.1;1.1)	2.8 (0.6;13.5)	2.5 (0.5;13.8)	2.5 (0.7;9.1)
**Caloric drinks**				
ParEdu High	1.4 (0.5;3.8)	4.5 (1.3;15.2)	0.7 (0.2;2.0)	2.2 (0.9;5.6)
Income med	0.3 (0.1;0.8)	0.8 (0.3;2.5)	0.8 (0.3;2.4)	0.8 (0.3;2.1)
Income high	1.0 (0.3;2.8)	0.6 (0.2;2.1)	1.2 (0.4;4.0)	1.3 (0.5;3.6)
School high	0.8 (0.3;2.2)	0.8 (0.3;2.3)	1.3 (0.5;3.7)	0.5 (0.2;1.1)
Puberty yes	1.7 (0.5;5.5)	2.4 (0.6;9.9)	2.6 (0.6;10.8)	1.0 (0.3;3.6)
BMI	1.0 (0.9;1.2)	1.0 (0.8;1.2)	0.9 (0.7;1.1)	1.0 (0.9;1.2)
Sed high	2.0 (0.5;8.0)	3.0 (0.7;12.3)	1.1 (0.3;4.3)	1.7 (0.6;5.0)
**Tea [ml/d]**				
ParEdu High	1.0 (0.4;2.8)	1.5 (0.4;5.0)	1.3 (0.4;4.5)	1.6 (0.7;3.9)
Income med	0.6 (0.2;1.8)	1.1 (0.4;3.4)	1.2 (0.4;3.8)	0.9 (0.4;2.3)
Income high	1.1 (0.4;3.2)	1.1 (0.3;3.4)	1.0 (0.3;3.4)	0.5 (0.2;1.5)
School high	3.0 (1.2;7.5)	0.5 (0.1;1.6)	0.5 (0.1;1.5)	0.5 (0.2;1.1)
Puberty yes	0.3 (0.0;2.8)	1.3 (0.3;5.4)	0.7 (0.1;3.7)	0.7 (0.3;2.1)
BMI	0.9 (0.8;1.1)	1.0 (0.8;1.2)	0.8 (0.7;1.0)	0.9 (0.8;1.1)
Sed high	1.2 (0.4;4.1)	0.8 (0.2;3.8)	1.3 (0.4;4.9)	1.0 (0.3;3.4)
**Water [ml/d]**				
ParEdu High	1.4 (0.5;3.6)	1.0 (0.4;2.7)	1.1 (0.4;3.4)	1.0 (0.4;2.6)
Income med	0.4 (0.2;1.2)	2.4 (0.8;7.2)	1.6 (0.5;5.1)	1.2 (0.4;3.3)
Income high	1.0 (0.4;2.6)	2.1 (0.6;7.4)	1.1 (0.3;4.3)	1.1 (0.4;3.3)
School high	2.7 (1.0;7.4)	0.8 (0.3;2.0)	1.1 (0.4;3.0)	0.7 (0.3;1.8)

Puberty yes	0.4 (0.1;2.0)	1.6 (0.5;5.3)	1.1 (0.3;4.7)	1.1 (0.3;3.8)
BMI	1.2 (1.0;1.4)	1.1 (0.9;1.4)	1.2 (1.0;1.5)	0.8 (0.7;1.0)
Sed high	0.5 (0.1;1.9)	1.2 (0.3;4.0)	1.1 (0.3;4.0)	0.9 (0.2;3.8)
**Protein[5]**				
ParEdu High	0.8 (0.3;1.9)	1.0 (0.4;2.5)	0.5 (0.2;1.6)	1.7 (0.7;4.2)
Income med	0.9 (0.4;2.4)	0.8 (0.3;2.2)	2.2 (0.7;7.5)	0.9 (0.3;2.3)
Income high	0.8 (0.3;2.0)	0.7 (0.2;2.1)	2.2 (0.6;8.4)	0.6 (0.2;1.7)
School high	0.8 (0.3;2.0)	2.4 (0.9;6.1)	2.4 (0.8;7.1)	0.5 (0.2;1.2)
Puberty yes	1.8 (0.5;7.0)	1.5 (0.4;6.5)	1.9 (0.4;9.3)	0.8 (0.3;2.2)
BMI	1.1 (0.9;1.3)	1.0 (0.8;1.2)	1.0 (0.8;1.2)	1.0 (0.9;1.2)
Sed high	1.0 (0.3;3.3)	1.9 (0.5;6.9)	2.6 (0.7;10.5)	1.1 (0.3;3.8)
**Fat**				
ParEdu High	0.6 (0.2;1.5)	0.4 (0.1;1.3)	0.8 (0.3;2.2)	0.9 (0.3;2.2)
Income med	1.9 (0.7;4.8)	0.8 (0.2;2.3)	1.3 (0.5;3.6)	1.0 (0.4;2.7)
Income high	2.9 (1.0;8.5)	0.8 (0.3;2.7)	1.1 (0.4;3.2)	1.6 (0.6;4.7)
School high	0.8 (0.3;2.1)	1.6 (0.5;4.6)	1.1 (0.4;2.9)	1.1 (0.5;2.7)
Puberty yes	0.7 (0.2;2.8)	2.3 (0.6;8.6)	1.3 (0.4;4.8)	0.7 (0.2;2.3)
BMI	0.9 (0.7;1.1)	0.8 (0.7;1.0)	0.9 (0.8;1.1)	1.1 (0.9;1.3)
Sed high	0.8 (0.2;2.9)	8.1 (1.3;48.6)	7.5 (1.3;42.0)	1.5 (0.5;4.7)
**Carbohydrate**				
ParEdu High	1.9 (0.7;4.8)	1.6 (0.6;4.6)	0.9 (0.3;2.7)	0.5 (0.2;1.4)
Income med	0.8 (0.3;2.1)	1.9 (0.6;5.6)	0.6 (0.2;1.8)	2.2 (0.8;6.0)
Income high	1.1 (0.4;3.0)	1.9 (0.6;6.4)	0.6 (0.2;2.2)	2.4 (0.9;6.6)
School high	0.9 (0.4;2.3)	1.6 (0.5;4.4)	1.6 (0.5;5.0)	0.6 (0.2;1.6)
Puberty yes	0.9 (0.3;2.5)	1.6 (0.4;6.4)	1.7 (0.4;7.3)	0.4 (0.1;1.8)
BMI	1.1 (0.9;1.3)	0.9 (0.8;1.1)	0.9 (0.7;1.1)	0.9 (0.8;1.1)
Sed high	4.3 (1.0;17.7)	7.0 (1.2;41.0)	9.4 (1.7;50.9)	0.5 (0.2;1.8)
**n6PUFA**				
ParEdu High	0.8 (0.3;2.2)	1.1 (0.4;3.1)	1.9 (0.6;5.9)	2.3 (1.0;5.5)
Income med	1.2 (0.4;3.5)	1.1 (0.4;3.1)	1.4 (0.4;4.3)	0.8 (0.3;2.1)
Income high	1.4 (0.5;4.4)	0.6 (0.2;2.3)	2.0 (0.6;7.1)	1.1 (0.4;2.9)
School high	1.7 (0.7;4.5)	0.6 (0.2;1.7)	1.0 (0.3;3.0)	0.9 (0.4;2.1)
Puberty yes	2.2 (0.6;8.5)	0.6 (0.1;2.5)	0.4 (0.1;2.1)	0.6 (0.2;2.0)
BMI	1.1 (0.9;1.3)	0.8 (0.6;1.0)	0.8 (0.7;1.0)	1.1 (0.9;1.3)
Sed high	1.8 (0.6;5.8)	0.4 (0.1;1.8)	0.7 (0.2;2.6)	0.9 (0.2;3.4)
**Retinol [ml/d]**				
ParEdu High	1.3 (0.5;3.5)	2.3 (0.8;6.4)	1.5 (0.5;4.1)	1.0 (0.4;2.4)
Income med	0.7 (0.3;1.8)	2.5 (0.8;7.7)	1.0 (0.4;3.0)	0.7 (0.3;1.8)
Income high	0.5 (0.2;1.2)	5.1 (1.5;17.2)	1.1 (0.3;3.8)	0.7 (0.2;2.4)
School high	0.7 (0.2;1.7)	0.5 (0.2;1.4)	1.6 (0.6;4.3)	1.0 (0.4;2.7)
Puberty yes	1.0 (0.3;3.2)	8.3 (1.3;52.2)	5.0 (0.8;30.4)	0.7 (0.2;2.0)
BMI	1.0 (0.8;1.2)	1.0 (0.9;1.2)	1.1 (1.0;1.4)	1.1 (0.9;1.3)
Sed high	1.0 (0.4;2.8)	1.4 (0.3;6.9)	0.8 (0.2;4.0)	0.5 (0.1;1.6)
**Beta-Carotene [ml/d]**				
ParEdu High	0.7 (0.3;1.8)	0.5 (0.2;1.4)	0.6 (0.2;2.0)	1.9 (0.7;5.0)
Income med	0.9 (0.3;2.4)	0.8 (0.3;2.1)	0.5 (0.2;1.5)	0.9 (0.3;2.4)
Income high	1.2 (0.4;3.2)	0.9 (0.3;3.1)	0.7 (0.2;2.4)	0.6 (0.2;1.6)
School high	1.7 (0.7;4.2)	1.5 (0.5;4.1)	1.8 (0.6;5.2)	1.0 (0.4;2.7)
Puberty yes	0.7 (0.2;2.7)	0.6 (0.2;1.9)	0.8 (0.2;2.7)	2.1 (0.6;7.8)
BMI	1.0 (0.9;1.2)	1.2 (1.0;1.5)	1.2 (0.9;1.4)	0.8 (0.6;1.0)
Sed high	0.5 (0.2;1.4)	0.6 (0.1;2.5)	1.3 (0.4;4.9)	2.1 (0.4;10.4)
**Alpha tocopherol [ml/d]**				
ParEdu High	1.4 (0.6;3.5)	1.3 (0.4;3.9)	0.8 (0.3;2.5)	1.8 (0.7;4.7)
Income med	1.1 (0.4;2.8)	2.1 (0.7;6.8)	1.0 (0.3;3.1)	0.6 (0.2;1.6)
Income high	1.3 (0.5;3.6)	1.4 (0.4;4.9)	1.5 (0.4;5.1)	0.6 (0.2;1.6)
School high	0.8 (0.3;1.8)	0.6 (0.2;1.7)	0.6 (0.2;1.8)	1.2 (0.4;3.2)

Puberty yes	1.0 (0.3;3.0)	0.6 (0.1;4.1)	1.0 (0.2;6.0)	1.9 (0.6;5.8)
BMI	0.9 (0.8;1.1)	1.0 (0.8;1.2)	0.9 (0.7;1.1)	1.1 (0.9;1.3)
Sed high	2.3 (0.7;7.8)	0.1 (0.0;1.3)	1.5 (0.4;5.2)	6.4 (1.2;33.4)

[1]Odds ratio (95% CI); [2] Logistic regression (increase vs. tracking in lowest tertile). [3]Multinomial logistic regression (increase or decrease vs. tracking in medium tertile), [4]Logsitic regression (decrease vs. tracking in highest tertile); [5]Multinomial regression not adjusted for diet change; [6]Multinomial regression not adjusted for pubertal onset; ParEdu high: parental education (high vs. low); Income med/high: family income (medium/high vs. low); ChildEdu high: child education (high vs. low); Puberty yes: pubertal onset at baseline (yes vs. no); Screen high: screen-time at baseline (high vs. low).*p-value < 0.0083 (Bonferroni correction for multiple testing: 0.05/6)
